# Microevolution of *Candida albicans* in Macrophages Restores Filamentation in a Nonfilamentous Mutant

**DOI:** 10.1371/journal.pgen.1004824

**Published:** 2014-12-04

**Authors:** Anja Wartenberg, Jörg Linde, Ronny Martin, Maria Schreiner, Fabian Horn, Ilse D. Jacobsen, Sabrina Jenull, Thomas Wolf, Karl Kuchler, Reinhard Guthke, Oliver Kurzai, Anja Forche, Christophe d'Enfert, Sascha Brunke, Bernhard Hube

**Affiliations:** 1 Department of Microbial Pathogenicity Mechanisms, Leibniz Institute for Natural Product Research and Infection Biology – Hans Knoell Institute Jena (HKI), Jena, Germany; 2 Research Group Systems Biology & Bioinformatics, Leibniz Institute for Natural Product Research and Infection Biology – Hans Knoell Institute Jena (HKI), Jena, Germany; 3 Septomics Research Center, Friedrich Schiller University and Leibniz Institute for Natural Product Research and Infection Biology –Hans Knoell Institute, Jena, Germany; 4 Research Group Microbial Immunology, Leibniz Institute for Natural Product Research and Infection Biology – Hans Knoell Institute Jena (HKI), Jena, Germany; 5 Medical University Vienna, Max F. Perutz Laboratories, Department of Medical Biochemistry, Vienna, Austria; 6 Department of Biology, Bowdoin College, Brunswick, Maine, United States of America; 7 Institut Pasteur, Unité Biologie et Pathogénicité Fongiques, Département Génomes et Génétique, Paris, France; 8 INRA, USC2019, Paris, France; 9 Integrated Research and Treatment Center, Sepsis und Sepsisfolgen, Center for Sepsis Control and Care (CSCC), Universitätsklinikum Jena, Germany; 10 Friedrich Schiller University, Jena, Germany; University College Dublin, Ireland

## Abstract

Following antifungal treatment, *Candida albicans*, and other human pathogenic fungi can undergo microevolution, which leads to the emergence of drug resistance. However, the capacity for microevolutionary adaptation of fungi goes beyond the development of resistance against antifungals. Here we used an experimental microevolution approach to show that one of the central pathogenicity mechanisms of *C. albicans*, the yeast-to-hyphae transition, can be subject to experimental evolution. The *C. albicans cph1*Δ/*efg1*Δ mutant is nonfilamentous, as central signaling pathways linking environmental cues to hyphal formation are disrupted. We subjected this mutant to constant selection pressure in the hostile environment of the macrophage phagosome. In a comparatively short time-frame, the mutant evolved the ability to escape macrophages by filamentation. In addition, the evolved mutant exhibited hyper-virulence in a murine infection model and an altered cell wall composition compared to the *cph1*Δ/*efg1*Δ strain. Moreover, the transcriptional regulation of hyphae-associated, and other pathogenicity-related genes became re-responsive to environmental cues in the evolved strain. We went on to identify the causative missense mutation via whole genome- and transcriptome-sequencing: a single nucleotide exchange took place within *SSN3* that encodes a component of the Cdk8 module of the Mediator complex, which links transcription factors with the general transcription machinery. This mutation was responsible for the reconnection of the hyphal growth program with environmental signals in the evolved strain and was sufficient to bypass Efg1/Cph1-dependent filamentation. These data demonstrate that even central transcriptional networks can be remodeled very quickly under appropriate selection pressure.

## Author Summary

Pathogenic microbes often evolve complex traits to adapt to their respective hosts, and this evolution is ongoing: for example, microorganisms are developing resistance to antimicrobial compounds in the clinical setting. The ability of the common human pathogenic fungus, *Candida albicans*, to switch from yeast to hyphal (filamentous) growth is considered a central virulence attribute. For example, hyphal formation allows *C. albicans* to escape from macrophages following phagocytosis. A well-investigated signaling network integrates different environmental cues to induce and maintain hyphal growth. In fact, deletion of two central transcription factors in this network results in a mutant that is both nonfilamentous and avirulent. We used experimental evolution to study the adaptation capability of this mutant by continuous co-incubation within macrophages. We found that this selection regime led to a relatively rapid re-connection of signaling between environmental cues and the hyphal growth program. Indeed, the evolved mutant regained the ability to filament and its virulence *in vivo*. This bypass of central transcription factors was based on a single nucleotide exchange in a gene encoding a component of the general transcription regulation machinery. Our results show that even a complex regulatory network, such as the transcriptional network which governs hyphal growth, can be remodeled via microevolution.

## Introduction

The incidence of invasive fungal infections has steadily increased within the past decades, largely because of a growing population of susceptible individuals, reflecting the progress of modern medicine in prolonging life even with severe underlying diseases and the increasing rate of immuno-deficient patients. One of the most frequently isolated fungi is *Candida albicans*, an ubiquitous and normally harmless commensal of the alimentary tract and mucocutaneous membranes. As an opportunistic pathogen, it can cause superficial infections like oropharyngeal candidiasis, especially in HIV patients, as well as life-threatening systemic infections with mortality rates up to 40%, even with current antifungal treatment options [1].

The transition from the commensal to a pathogenic state depends on the microbiota, the host response, and *C. albicans* activities, such as adhesion, secretion of hydrolases, metabolic adaptation, biofilm formation and, importantly, morphological plasticity, which includes the yeast-to-filament transition [2]–[7]. To survive and thrive in the many different niches inside the host, *C. albicans* must be able to adapt to changing environments and different stresses. In the short term, this occurs primarily by changes in gene expression and translation, and via post-translational modifications, but ultimately microevolutionary processes will play an important role. As a prominent example, White *et al.* [8] have shown that microevolution is the driving force behind the emergence of antifungal drug resistance. They demonstrated the *de novo* appearance of fluconazole resistance in evolving *C. albicans* strains *in vivo* [8]. Furthermore, clinical isolates generally exhibit large genetic variations, and microevolution can be observed both *in vitro* and *in vivo* [9], [10], indicating that this process plays an important role in host-pathogen interactions. Therefore, microevolution provides a source of variation for the adaptive response of *C. albicans* to challenging (host) environments.

Different mechanisms account for the generation of new genotypic variants, including point mutations, amplification or deletion of chromosomal segments, chromosomal translocation or inversion, and whole chromosome aneuploidy. These genetic variations can affect expression of single genes or the structure of their encoded proteins as well as whole transcriptional networks via a mechanism known as transcriptional rewiring. In this process, the interaction between promoter regions and their corresponding regulators can be switched to different pairings, which in turn cause new connections to be formed between a signal and a transcriptional response [11], [12]. Whereas many studies have explored the underlying mechanisms of drug resistance, the role that microevolution plays in host-pathogen interactions has rarely been investigated: Forche *et al.* [13] found that a *C. albicans* strain, passaged through a mouse host, responded by undergoing chromosome-level genetic variations, which were sufficient to generate new variants of *C. albicans*.

The yeast-to-hyphae transition of *C. albicans* is central for pathogenicity [14], [15]. Filamentation plays a pivotal role for adhesion to, invasion into and damage of epithelial and endothelial cells [2], [16], [17]. Upon internalization by macrophages, *C. albicans* induces host cell death by triggering pyroptosis, a form of programmed cell death [18], [19]. However, later in the infection process the yeast-to-hyphae transition contributes to escape from the phagosome [19], [20]. Morphology also plays a key role in host recognition [21]. Given the importance of morphology of *C. albicans* for pathogenicity, it is not surprising that the yeast-to-filament transition is induced by a wide range of environmental factors and conditions like high pH, host body temperature, CO_2_, starvation and presence of serum, all of which act via several signaling pathways. Among them, the cAMP-dependent protein kinase A (cAMP-PKA) and the mitogen-activated protein kinase (MAPK) pathways, which target the transcription factors Efg1 and Cph1, respectively, play a central role in hyphal formation [22], [23]. This is demonstrated by a *cph1*Δ/*efg1*Δ double mutant, which is unable to form hyphae under almost all hyphae-inducing conditions *in vitro* (except agar embedded conditions) and which is probably the most commonly used mutant of *C. albicans* in a wide range of experiments [14], [15], [22], [24].

Due to the central role of the yeast-to-filament transition for *C. albicans* virulence, we used the *cph1*Δ/*efg1*Δ double mutant as a model for evolutionary adaptation. To this end, we performed a series of co-culture passages of this mutant with macrophages. We expected that the hostile environment of the phagosome imposes a high selective pressure on the fungus favoring either intracellular adaptation or return to filamentation in order to escape. We performed phenotypic, transcriptomic and genomic analyses of the pre- and post-passaged strains to elucidate the degree of genetic plasticity of *C. albicans* when facing host stresses. We show that adaptation to macrophages leads to distinct phenotypic differences between the pre- and post-passaged strains with regained filamentation in the latter. As the causative mutation, we identified a heterozygous, non-synonymous single nucleotide exchange in the gene *SSN3*, which encodes the cyclin-dependent kinase of a regulatory module of the Mediator complex. Our results demonstrate that the regulation of the morphological switch in *C. albicans* can be subject to microevolution.

## Results

### Experimental microevolution causes a reversion of the nonfilamentous phenotype of the *cph1*Δ/*efg1*Δ mutant strain

To determine the ability of *C. albicans* to adapt to stresses inside phagocytes and to test the adaptability of the hyphal regulatory network, we first screened for mutants which are unable to escape from macrophages via filamentation response. We tested multiple *C. albicans* deletion strains with known defects in hyphal formation: strains lacking *RAS1*, *RIM101*, *DFG16*, *TEC1*, *HGC1*, *EED1*, or *UME6* and the avirulent double deletion mutant lacking *CPH1* and *EFG1* [22]. Of these, only the *cph1*Δ/*efg1*Δ double mutant was completely unable to escape from macrophages even after 24 hours, while all other mutants still formed filaments inside the host cell and pierced the phagocyte membrane to some extent (S1A Figure). Microscopy with FITC-labeled *cph1*Δ/*efg1*Δ cells revealed that these cells were viable and still able to replicate in the yeast form after ingestion by macrophages (S1B Figure).

Therefore, we chose the *cph1*Δ/*efg1*Δ strain for the following microevolution experiment. Cells of the murine macrophage cell line J774A.1 were infected with the *cph1*Δ/*efg1*Δ double mutant at a macrophage-to-fungal ratio of 2∶1 and co-incubated. Every 24 hours, non-phagocytosed cells were removed and macrophages were lysed to harvest the phagocytosed cells. A defined fraction of this population was then transferred to a fresh macrophage population.

After 19 passages, a significant morphological alteration became visible, as several phagocytosed cells started to form filaments. These filamenting cells became fixed in the population after additional 23 rounds of co-incubation. This morphologically distinct variant, evolutionary derived from the *cph1*Δ/*efg1*Δ mutant, was termed Evo. The absence of *CPH1* and *EFG1* in the Evo strain was verified by Southern blot analysis (S1C Figure). To exclude temporary or epigenetic effects, the Evo strain was repassaged daily in liquid rich (YPD) medium without any selection pressure by host cells for 14 passages. The phenotype remained stable and no reversal was detected.

**Figure 1:**
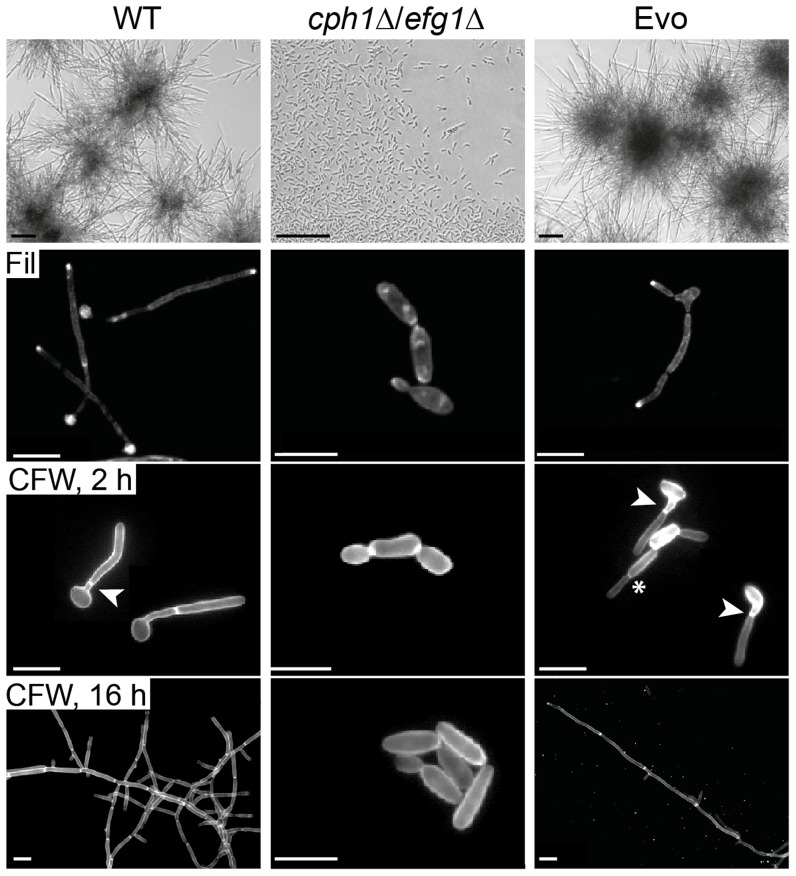
Co-incubation with macrophages led to regained filamentation in the *cph1*Δ/*efg1*Δ strain. Morphology of wild type (WT), *cph1*Δ/*efg1*Δ and Evo strain after 18 h of incubation in DMEM+10% FBS at 37°C and 5% CO_2_ demonstrate the re-appearance of filamentation in the Evo strain (scale bar: 100 µm, upper panel). All strains were grown for 4 h for filipin (Fil) staining, and for 2 h or 16 h for calcofluor white (CFW) staining on cover slips, and analyzed by fluorescence microscopy (scale bar: 10 µm, lower panels). Arrow heads highlight septa (true hyphae), while asterisks indicate constrictions (pseudohyphae).

To test whether the regained ability to form filaments was restricted to macrophage interactions or observed under additional hypha-inducing conditions, we analyzed the morphology of the Evo strain in the absence of host cells. In the cell culture medium DMEM with 10% serum at 37°C and 5% CO_2_, clear filament formation of the Evo strain, but not the *cph1*Δ/*efg1*Δ strain, was observed (Fig. 1). Filamentous growth is associated with highly polarized ergosterol inclusion in membranes, which can be visualized by filipin staining [25]. As shown in Fig. 1, Evo cells grown in the presence of serum exhibited intense filipin staining at the filament tips, equal to the wild type cells. Consistent with the defect in polarized growth, the *cph1*Δ/*efg1*Δ strain showed a more uniform filipin staining. Staining with calcofluor white for morphology analyses showed the expected true hyphae for the wild type and elongated yeasts for the *cph1*Δ/*efg1*Δ strain (Fig. 1). Interestingly, the Evo strain showed heterogeneous cell morphologies, i.e. a mixture of pseudohyphae with constrictions at the septa and true hyphae with parallel-sided walls (Fig. 1). The percentage of the different morphological forms was quantified using the morphological index (MI) [26] of individual cells after 4 and 12 hours of growth in serum (S2A Figure). The MI for *cph1*Δ/*efg1*Δ was <2.5 at both time points, indicating yeast morphology. In contrast, most cells of the Evo strain grew as pseudohyphae (MI 2.5–3.4) after 4 hours, while after 12 hours true hyphae were evident (MI>3.4) in approx. 50% of the population. Both morphologies will be referred to as filaments.

We then tested different classical hyphae-induction media for *C. albicans* to assess the extent of phenotype reversal to wild type morphology. In response to serum-containing YPD medium with 5% CO_2_, the Evo strain initially formed filaments but switched back to yeast growth much earlier than the wild type (S2B Figure). Filamentation (mainly pseudohyphae) also occurred in response to the amino sugar N-acetyl-D-glucosamine as sole carbon source and 5% CO_2_ (S2B Figure). Finally, cells of the Evo strain were incubated in serum-containing water at 37°C in atmospheric air. Again, stable filamentation was induced, demonstrating that high CO_2_ is not absolutely necessary for filamentation of the Evo strain (S2B Figure). In embedded media at 23°C (S2C Figure.), deletion of *EFG1* causes a hyperfilamentous phenotype [24]. Accordingly, the *cph1*Δ/*efg1*Δ strain was hyperfilamentous under these conditions. Interestingly, while cells of the Evo strain displayed an even more pronounced hyperfilamentous phenotype, it did not undergo filamentation on solid medium at 37°C, as seen in the *cph1*Δ/*efg1*Δ strain (S2D Figure).

In conclusion, our microevolution experiment led to the regained ability of filamentous growth in the *cph1*Δ/*efg1*Δ mutant in response to a diverse range of hyphae-inducing conditions, indicating that microevolutionary events had enabled this strain to bypass the dependency on Cph1 and Efg1 for filamentation in these media.

### The Evo strain regained virulence potential

**Figure 2:**
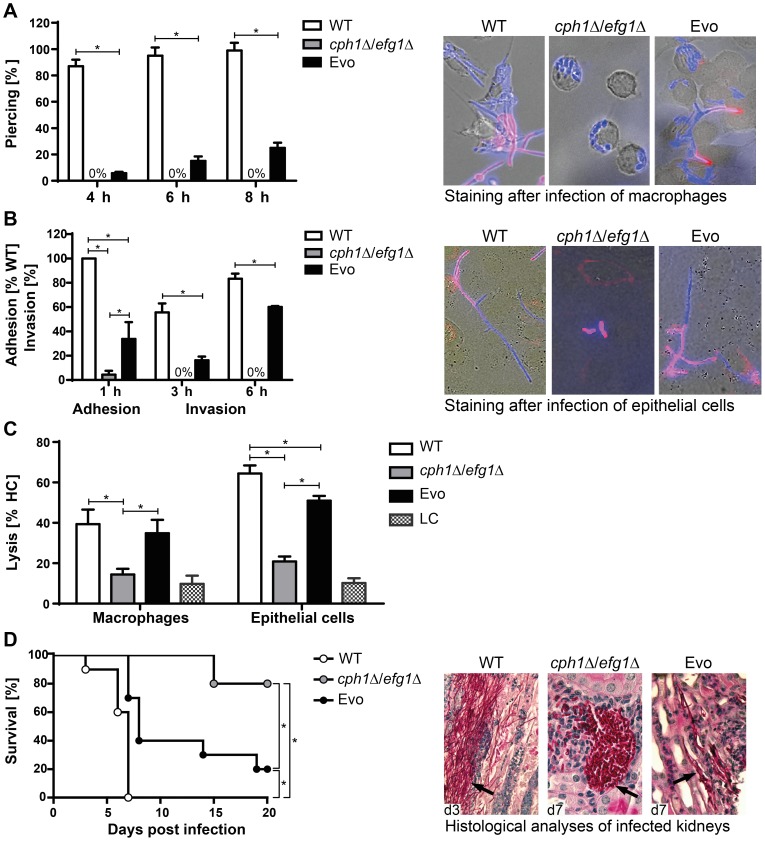
Characterization of Evo strain interaction with host cells and virulence potential. (**A**) Escape of *C. albicans* cells by piercing of macrophages (J774A.1) after different timepoints (left). Micrographs of strains after 6 h of co-incubation with J774A.1 cells (right). Intracellular *C. albicans* appears blue (CFW), extracellular section of the cells red (Concanavalin A, ConA). Cells of the *cph1*Δ/*efg1*Δ strain cannot escape from macrophages, while Evo cells regained this property during the evolution experiment. (**B**) Adhesion to and invasion of oral epithelial cells (TR-146). Adhesion values are given as percentage of adherent WT cells (left). Micrographs show filamentation of *C. albicans* WT and Evo strains after 6 h of incubation with TR-146 cells (right). The regained ability to filament enabled the Evo strain to invade epithelial cells. Staining was performed as described in (A). (**C**) Damage to macrophages and epithelial monolayers, determined by lactate dehydrogenase (LDH) assay after 32 h of co-incubation (LC = low control, medium only). WT and Evo strain, but not the *cph1*Δ/*efg1*Δ strain caused clear damage to both cell types. For piercing, adhesion, invasion and cell damage assay results are given as mean+SD of three independent experiments (*p<0.05). (**D**) Survival of BALB/c mice challenged intravenously (left; n = 10/strain). Nearly all mice infected with the Evo strain succumbed to the infection, while almost all animals infected with *cph1*Δ/*efg1*Δ strain survived (*p<0.05). PAS-hematoxylin-stained kidney sections from different days (d) post challenge (right) show fungal cells (arrows) either in the filamentous form (WT and Evo strain) or yeast form (*cph1*Δ/*efg1*Δ strain).

Filamentous growth is an important contributing factor for the escape from macrophages. We therefore determined the amount of Evo cells that escaped from macrophages by piercing through their membranes after 4 h, 6 h and 8 h of co-incubation (Fig. 2A). Both Evo and wild type, but not the *cph1*Δ/*efg1*Δ double mutant, were able to escape from macrophages. However, the piercing rate of the Evo strain was significantly lower than for the wild type at all time points. After 8 h of co-incubation nearly all wild type cells had escaped from the macrophages, but only about 25% of Evo cells. The delay in filamentation and the presence of pseudohyphae in the Evo strain may explain these differences. Next, we assessed the fungus' ability to invade oral epithelial cells. Invasion requires previous adhesion, and the *cph1*Δ/*efg1*Δ strain was almost entirely unable to adhere to epithelial cells (Fig. 2B). Adhesion of the Evo strain was still reduced compared to the wild type, but significantly higher than for the double mutant (Fig. 2B). This is reflected by the invasion capacity of the Evo strain, which was significantly lower than the wild type strain, but substantially higher than the *cph1*Δ/*efg1*Δ strain. Finally, we also investigated the potential of the Evo strain to damage macrophages and epithelial cells by measuring the release of lactate dehydrogenase (LDH). After 32 hours of co-incubation, the Evo strain had damaged macrophages to the same extent as the wild type strain, and epithelial cells to a significantly higher degree than the *cph1*Δ/*efg1*Δ strain (Fig. 2C).

The Evo strain had thus regained abilities putatively relevant for systemic infections. Hence, the virulence of the Evo strain was tested in a murine model of hematogenously disseminated candidiasis. Survival was monitored over a period of 21 days. As predicted, mice infected with the Evo strain showed an intermediate and significantly different survival rate compared to mice infected with the wild type and *cph1*Δ/*efg1*Δ strains (Fig. 2D). Histological examination of kidneys from infected animals revealed that the Evo strain retained its filamentous morphology *in vivo*, even though filaments formed by the Evo strain were shorter than by the wild type, and invasion into deeper layers of the kidney tissue was less pronounced (Fig. 2D).

In summary, the evolved changes in response to macrophages enabled the Evo strain not only to form filaments *in vitro*, but also in contact with host cells, which correlated with a higher virulence potential both *in vitro* and *in vivo*.

### The Evo strain expresses hyphal-associated genes and responds to farnesol

**Figure 3:**
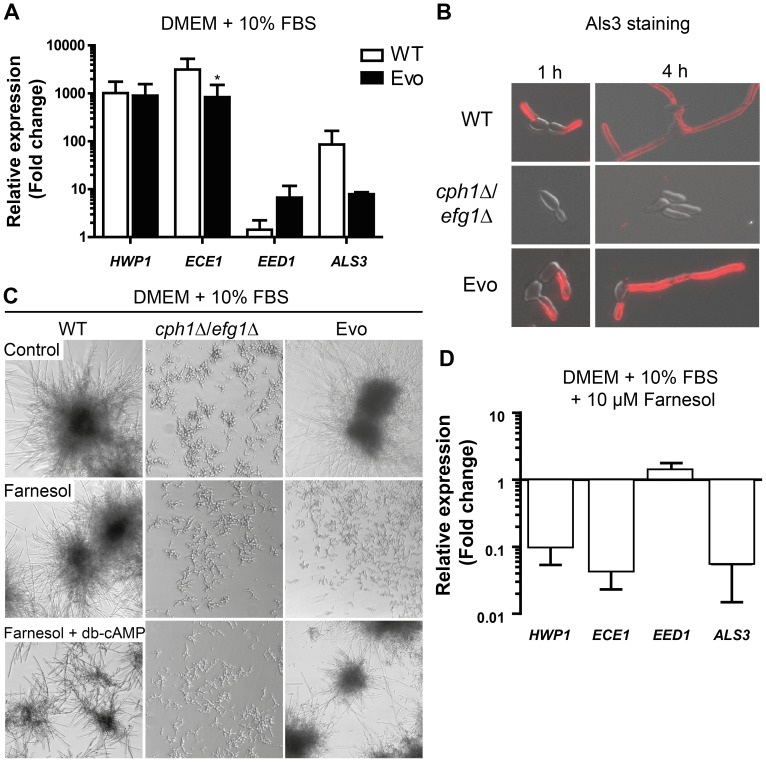
Analysis of hyphae-associated gene expression, Als3 surface expression and response to farnesol. (**A**) The Evo strain expresses hyphae-associated genes after growth for 1 h at 37°C at 5% CO_2_ on a plastic surface similar to WT. Relative gene expression of filament-inducing conditions was compared to yeast promoting conditions (YPD, 30°C) for three independent experiments. Expression was normalized against three housekeeping genes (*ACT1*, *EFB1* and *PMA1*) and data are shown as mean+SD of three biological experiments (*p<0.05). (**B**) Immunofluorescence micrographs of cells immuno-stained for Als3 after growth in DMEM+10% FBS at 37°C and 5% CO_2_ on cover slips. Wild type (WT) and Evo cells are Als3-positive, while *cph1*Δ/*efg1*Δ cells show no signal (representative samples). (**C**) Morphogenetic response to farnesol treatment alone or in combination with exogenous dibutyryl-cyclic AMP (db-cAMP). All strains were exposed to either methanol (control), 1 µM farnesol or 1 µM farnesol+10 mM db-cAMP and incubated at 37°C and 5% CO_2_ for 18 h (representative pictures from three independent experiments are shown). Note that in Evo cells inhibition of filamentation by farnesol treatment was completely abrogated when db-cAMP was added. (**D**) Repression of hyphae-associated gene expression in the Evo strain by 10 µM farnesol. Expression was normalized against three housekeeping genes (*ACT1*, *EFB1* and *PMA1*). The fold change in expression relative to filament-inducing conditions alone is shown as mean+SD of three biological experiments.

Hyphal-associated virulence of *C. albicans* is not only due to filamentation *per se*, but also to the expression of hyphae-associated genes. In order to monitor the expression of typical hyphae-associated genes in the Evo strain, we measured the mRNA levels of *HWP1*, *ECE1* and *ALS3*, all encoding hyphal cell surface proteins, and of *EED1*, a gene that is associated with hyphal cell elongation [27]. An upregulation of all four genes in the Evo strain was confirmed by qRT-PCR after 1 hour of incubation in DMEM+10% FBS at 37°C and 5% CO_2_ (Fig. 3A). *HWP1* expression was similar in the Evo and WT strain, whereas *ECE1* and *ALS3* were higher expressed in the WT strain, and *EED1* was more strongly upregulated in the Evo strain. Furthermore, we observed Als3 exposure on the surface of wild type and Evo cells by immunofluorescence, but not on the *cph1*Δ/*efg1*Δ strain (Fig. 3B). This regained cell-surface exposure of the Als3 adhesin [28] is in accordance with the increased adhesion potential of the Evo strain.

We were next interested if the filamentation program can be blocked by the quorum-sensing molecule farnesol. Very low concentrations (1 µM) of farnesol in the medium resulted in a complete repression of filament formation in the Evo strain, whereas wild type cells still formed hyphae (Fig. 3C). Consistently, farnesol treatment led to a dramatic repression of filament-associated gene expression (Fig. 3D). By addition of exogenous dibutyryl-cyclic AMP (db-cAMP) to the farnesol-containing medium, filamentation was rescued in the Evo strain (Fig. 3C). These data suggest a critical role for cAMP signaling in the filamentation process of the Evo strain.

### The Evo strain shows wild type levels of filamentation-associated transcription factor gene expression

**Figure 4:**
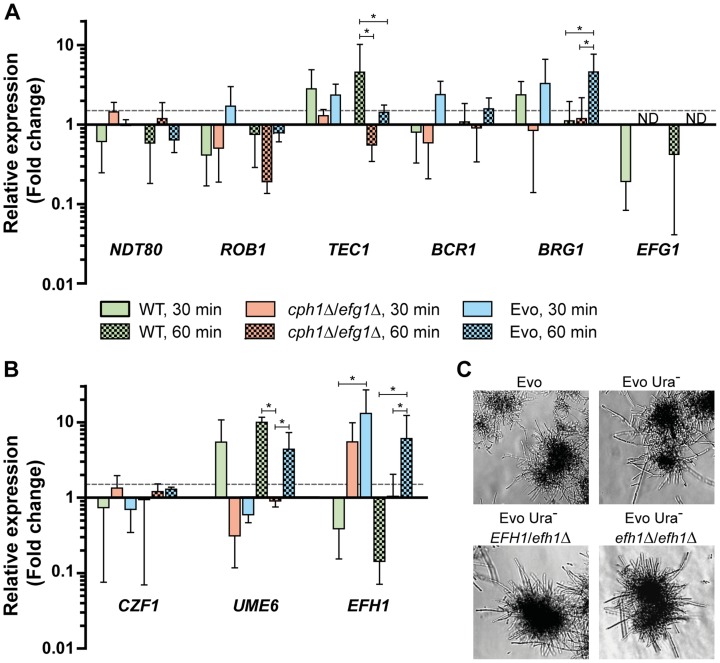
Expression of transcription factors under filament-inducing conditions. (**A+B**) Relative expression of nine central transcription factor genes in the analyzed strains after growth in DMEM+10% FBS at 37°C and 5% CO_2_ on a plastic surface. Fold change between filament-inducing and yeast promoting conditions (YPD, 30°C) is shown, normalized to three housekeeping genes (*ACT1*, *EFB1* and *PMA1*). Means+SD of n = 3 (dotted line indicates threshold at 1.5; *p<0.05). (**C**) Deletion of *EFH1* in the Evo strain did not affect hyphal growth. Cells were incubated for 18 h at 37°C and 5% CO_2_ in DMEM+10% FBS (representative pictures).

The yeast-to-filament regulatory network comprises many different transcription factors (TFs). The filament-associated biofilm formation is controlled by a network formed by Bcr1, Tec1, Brg1, Rob1, Ndt80 and Efg1 [29]. Efg1 positively regulates all other TF genes in this network except *ROB1*. We measured the transcription of these central TF genes at 30 min and 60 min after filament induction. As shown in Fig. 4A, we found an at least 1.5-fold upregulation of *ROB1* and *TEC1* after 30 min, and of *BCR1* and *BRG1* at both timepoints in the Evo strain. The wild type, however, showed only an increased expression of *TEC1* at both timepoints and of *BRG1* after 30 min. In contrast, most of these TF genes were down- or scarcely upregulated in the *cph1*Δ/*efg1*Δ strain (Fig. 4A).

Formation of wild type filaments is also regulated in part by *CZF1* under certain conditions [30]. An increased expression of *CZF1*, however, is not the cause for filamentation in the Evo strain. The *CZF1* mRNA levels under serum induction did not greatly differ from the mRNA level in the *cph1*Δ/*efg1*Δ strain (Fig. 4B). In addition, the mRNA level of *UME6*, a key TF gene necessary for the maintenance of filamentation [31], was upregulated in wild type cells at both time points but not in the *cph1*Δ/*efg1*Δ strain (Fig. 4B). Interestingly, *UME6* expression was more than 4-fold upregulated in the Evo strain after 60 min growth in serum-containing medium.

*C. albicans* possesses an *EFG1* homolog, *EFH1*, and overexpression of this gene is known to induce pseudohyphal growth. In addition, like *EFG1*, *EFH1* is involved in the regulation of expression of filament-associated genes [24]. We found that *EFH1* showed the strongest upregulation (7.4-fold) among the tested TF genes in the Evo strain. However, deletion of *EFH1* did not abolish filamentation of an Evo strain derivative (Fig. 4C). Hence, the filamentation phenotype of the Evo strain was not linked to this TF.

In summary, the Evo strain has regained most of the transcriptional hallmarks of filament production, including the upregulation of the central transcription factor genes *TEC1*, *BRG1* and *UME6*. The few discrepancies to the wild type may partially explain the remaining differences in morphology. However, the late-phase upregulation of *UME6* indicates that the filament maintenance of the Evo strain is similar to the wild type at the transcriptional level. Furthermore, the function of Efg1 was not replaced by Efh1 in the Evo strain.

### The cell wall defects of the *cph1*Δ/*efg1*Δ mutant are reverted in the Evo strain

**Figure 5:**
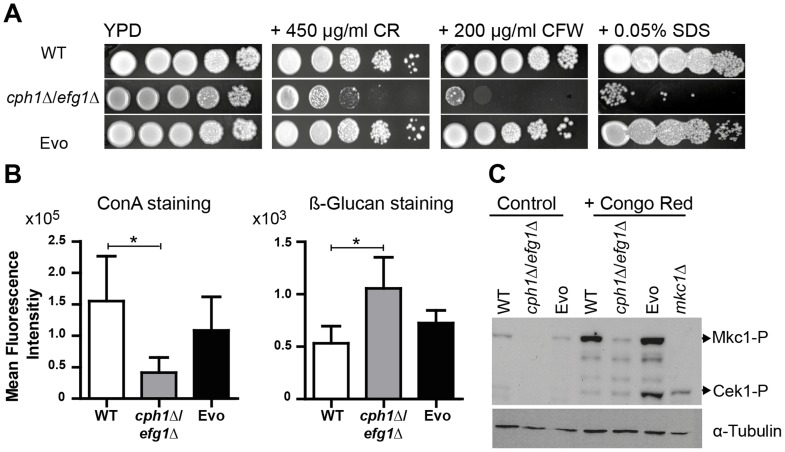
Microevolution led to decreased sensitivity of the Evo strain to different cell wall perturbing agents. (**A**) Resistance of analyzed strains against different cell wall stressors. The *cph1*Δ/*efg1*Δ strain was sensitive to all stresses while the Evo strain regained WT resistance (representative pictures of three experiments are shown). (**B**) Flow cytometry analysis of mannan and β-glucan exposure on the surface of live cells. Differences in fluorescence intensity between *cph1*Δ/*efg1*Δ strain and Evo strain point to an altered cell wall composition. Mean fluorescence intensity+SD of n = 3 (*p<0.05). (**C**) Western blot analysis to identify phosphorylated Mkc1 and Cek1 in *C. albicans* strains grown under non-stress conditions (control) or conditions of cell wall stress (450 µg/ml congo red) for 4 hours. Cell wall stress triggered phosphorylation of Mkc1 and Cek1 in the Evo strain, but not in the *cph1*Δ/*efg1*Δ strain. Tubulin served as loading control.

Our data indicated that the Evo strain regained the potential to produce hyphae, showed upregulation of transcription factor genes involved in filamentous growth and other hyphal associated genes, and regained a high virulence potential. The reduced virulence of the *cph1*Δ/*efg1*Δ strain is likely predominantly caused by the filamentation defects, however, Efg1 has also an important role in cell wall architecture [32] and the cell wall is essential for adhesion and invasive growth and thus for pathogenicity [33]. We therefore tested the Evo strain for cell wall defects by treatment with cell wall perturbing agents, i.e. congo red (CR), calcofluor white (CFW) and sodium dodecyl sulfate (SDS). As shown in Fig. 5A, the *cph1*Δ/*efg1*Δ strain was hypersensitive to all tested agents. In contrast, the Evo strain was as resistant as the wild type to CR and CFW, agents that disturb glucan and chitin architecture, respectively. The same phenotypic reversal was observed for the cell membrane disturbing agent SDS, suggesting a loose structure of the cell wall only in the *cph1*Δ/*efg1*Δ strain.

These results indicate that the altered cell wall composition of the *cph1*Δ/*efg1*Δ strain was at least partially restored in the Evo strain. We therefore stained exposed mannan and β-1,3-glucan with fluorescently labeled concanavalin A (ConA) and anti-β-1,3-glucan antibody, respectively (Fig. 5B). Quantification by FACS analysis displayed significantly reduced mannan and increased β-1,3-glucan signals on the surface of the *cph1*Δ/*efg1*Δ strain compared to the wild type strain. The Evo strain showed an intermediate mannan and wild type-like glucan exposure.

The two MAP kinases, Cek1 and Mkc1, become activated in wild type *C. albicans* upon treatment with cell wall disturbing agents [34]–[36]. After treatment with CR, both Cek1 and Mkc1 were phosphorylated in the Evo strain but not in the *cph1*Δ/*efg1*Δ strain (Fig. 5C). Unusually, only phosphorylated Mkc1 could be detected in the wild type strain, which may be due to changes in the CR treatment protocol compared to previous experiments performed by another group [34]. However, these results show that the Evo strain regained the ability to phosphorylate Mkc1 and Cek1 in response to cell wall stress.

### Global transcriptional analysis of the evolved strain by RNA-Seq

To gain insight into the regulatory program of filamentation in the absence of *CPH1* and *EFG1*, we performed gene expression analysis by RNA sequencing under hyphae- and non-hyphae-inducing conditions. Both, the *cph1*Δ/*efg1*Δ and the Evo strain, were analyzed with a sequencing depth sufficient to cover the genome 75–350×. Expression (RPKM≥1) was detected for 5,854 of the *C. albicans* open reading frames (94%), as well as for 561 nTARs (novel transcriptionally active regions, [37]), 67 small nuclear RNAs and for 24 tRNAs (see Materials and Methods for details and S2 Table for a complete list of all detected transcripts). Differential expression of selected genes was subsequently validated by qRT-PCR using biological replicates (S3A Figure).

After the transfer to filament-inducing conditions, 379 transcripts were significantly upregulated (≥2-fold, p<0.01) and 279 downregulated in the Evo strain. In the *cph1*Δ/*efg1*Δ strain, 255 transcripts were up- and 252 downregulated under the same condition. Within the group of upregulated transcripts, 209 genes were induced in both strains, while 46 transcripts were specifically induced in the *cph1*Δ/*efg1*Δ strain and 170 transcripts specifically in the Evo strain. 186 of the downregulated transcripts were repressed in both strains, whereas 66 and 93 transcripts were specifically repressed in the *cph1*Δ/*efg1*Δ and Evo strains, respectively (S3B Figure).

We investigated the expression of individual marker genes for filamentation [38] more closely (S3C Figure). As expected, all eight genes of the core filamentation response (*ALS3*, *ECE1*, *DCK1*, *HGT2*, *HWP1*, *IHD1*, *RBT1* and orf19.2457) were significantly upregulated in the Evo strain under filament-inducing conditions. Four of these genes (*ECE1*, *HWP1*, *IHD1* and *RBT1*) and further filament-associated genes, like *ALS1*, *BRG1* and *HGC1* were also upregulated in the non-filamenting *cph1*Δ/*efg1*Δ strain. Expression of filament-associated genes independent of any morphological transition has previously been described in the *cph1*Δ/*efg1*Δ mutant [38]–[40]. However, these genes were expressed at a significantly higher level in the Evo strain compared to the *cph1*Δ/*efg1*Δ strain under filament-inducing condition (S2 DS5 Table).

Overall, genes most highly expressed (≥5-fold) in the Evo strain under filament-inducing condition are mainly hyphal-associated genes (*HWP1*, *ECE1*, *ALS3*, *RBT1*, *FRG2*, *ALS1* and *IHD1*). Furthermore, the expression of *YWP1*, encoding a yeast-form cell wall protein, is downregulated in the Evo strain, while its expression did not change in the *cph1*Δ/*efg1*Δ strain. These results suggest that genes associated with *C. albicans* hyphae formation are also associated with filamentation of the Evo strain.

Upregulation (>1.5-fold, p<0.01; S2 DS1 and DS4 Table) of *DCK1*, *LMO1* and *CEK1*, which are required for filamentation under embedded conditions and for cell wall integrity [41], was found solely in the Evo strain. This provides a possible explanation for the hyper-filamentous phenotype under embedded conditions as well as the increased resistance to cell wall perturbants compared to the double mutant (Figs. 1+5).

To determine whether changes in the regulation of effector genes are reflected by an upregulation of specific TF genes, we also analyzed the expression levels of TF genes in the *cph1*Δ/*efg1*Δ and Evo strains under filament-inducing conditions in more depth (S2 DS7 Table). A significantly higher expression of 21 TF genes was shared by both strains, and only five TF genes were specifically upregulated in the *cph1*Δ/*efg1*Δ strain as compared to the levels in the Evo strain. Interestingly, 17 TF genes had significantly higher expression specifically in the Evo strain and not in *cph1*Δ/*efg1*Δ, including three genes known to be important hyphal morphogenesis regulators: *UME6* (in agreement with previous qRT-PCR results), *RIM101* and *HAC1*. Eight of the higher expressed TF genes in the Evo strain have unknown biological functions.

In the *cph1*Δ/*efg1*Δ strain, but not in the Evo strain, *CPH2*, *TEC1* and *ACE2*, which encode TFs involved in hyphal growth, were significantly downregulated under filament-inducing conditions. Finally, a significantly lower expression was observed for *NRG1* in the Evo strain, which codes for a repressor of hyphal development [42]. Hence, we scanned for Nrg1 binding sites (A/C)(A/C/G)C_3_T [43] in the putative promoter regions of genes specifically upregulated twofold in the Evo strain and detected the sequence motifs in 70% of these promoter regions (S2 DS8 Table). With this, the Nrg1 binding motif is statistically overrepresented in promoters of upregulated genes (p<0.01) when compared to promoters of all other genes. The downregulation of *NRG1* in the Evo strain may therefore facilitate expression of filament-associated genes and hence filament formation.

Further analyses indicated a significant upregulation of genes encoding for secreted aspartyl proteases (*SAP5*, *SAP6*, *SAP10*). In addition, significant differences in expression of genes associated with cell wall biosynthesis (*CHK1*, *KRE6*, *GLC3*, *MP65*, *ALG11* and *MNT2*), alkalinisation (*ACH1*) as well as of genes involved in glucose and galactose interconversion and uptake (*GAL10*, *GAL1*, *HGT*2, *HGT4*, *HGT12* and *GSY1*) were observed.

In summary, our transcriptional analysis indicated that serial passage through macrophages led to substantial alterations of the global transcriptional profile. The programs and pattern we found differed clearly from the *cph1*Δ/*efg1*Δ mutant, and resembled more the well-known programs of the wild type strain. This is concomitant with and likely correlated with the regained ability of the Evo strain to induce filaments and to induce damage to host cells *in vitro* and *in vivo*.

### Comparative whole genome re-sequencing identifies mutations potentially linked to Cph1/Efg1-independent filamentation

We went on to determine the genetic basis for the observed phenotypical differences. No obvious large-scale structural variations were detectable between the karyotypes of wild type, the *cph1*Δ/*efg1*Δ and Evo strains using pulsed field gel electrophoresis (PFGE; S4A Figure). To detect possible loss of heterozygosity (LOH) events [44], we analyzed four SNP-restriction fragment length polymorphism (RFLP) markers per chromosome [45]. No differences were detected between double mutant and Evo strain (S3 DS1 Table). Taken together, these data show that no gross chromosomal rearrangements have occurred in the Evo strain.

We re-sequenced the genomes of the Evo and the *cph1*Δ/*efg1*Δ strains to identify single nucleotide polymorphisms (SNP) that may have arisen during the microevolution experiment. Sequencing depth for *cph1*Δ/*efg1*Δ and Evo were 99× and 108× in average, respectively, with 98.8% of the *C. albicans* SC5314 reference genome covered in both cases. Comparison of both sequences revealed a chromosome 7 trisomy in the *cph1*Δ/*efg1*Δ strain, an aneuploidy that appears to have been lost during the evolution experiment (S4B Figure). This is also reflected by a 1.5× higher mean transcription level of genes on chromosome 7 in the *cph1*Δ/*efg1*Δ strain (S4C Figure). In addition, an amplification of *URA3* on chromosome 3 was observed. *URA3* was originally used as a marker to delete *CPH1* and *EFG1* in the *cph1*Δ/*efg1*Δ strain, and is now present in three copies in this mutant. The Evo strain contained 7–8 copies (S4B Figure). A qPCR analysis on isolated gDNA supported these findings (S4D Figure). PFGE and subsequent hybridization with a *URA3* specific probe further revealed that all copies were located on the same chromosome (S4E Figure). To exclude any possible contribution of multiple *URA3* gene copies to the filamentous phenotype, the Evo strain was cured from *URA3* with 5-fluoroorotic acid treatment [46]. This Evo Ura^−^ strain was still able to filament, showing that *URA3* copy number is not responsible for the filamentous phenotype (S4F Figure). Additionally, after re-introduction of a single *URA3* using the standard CIp10 plasmid at the *RPS10* locus [47], these strains exhibited the same adhesion, invasion and macrophage damage properties as their multi-*URA3* counterparts (S4G Figure). This indicates that the excessive *URA3* copies do not have an influence on classical virulence properties of *C. albicans*.

We observed a high number of SNPs in the *cph1*Δ/*efg1*Δ strain: altogether, 70,197 heterozygous and 3,156 homozygous SNPs were identified in *cph1*Δ/*efg1*Δ relative to the *C. albicans* SC5314 consensus reference genome (Assembly 21, [48]). Similarly, 72,315 heterozygous and 3,294 homozygous SNPs were identified in the Evo strain. These figures are consistent with those achieved when reads obtained by sequencing the genome of *C. albicans* SC5314 are aligned on the reference genome and reflect the high level of heterozygosity in *C. albicans* as well as putative sequencing errors and ambiguous positions in the reference genome (homozygous SNPs). After combining these sets and filtering, only 329 putative SNPs were found to distinguish the *cph1*Δ/*efg1*Δ and Evo strains. Notably, polymorphisms at 209 of these positions are observed in the genomes of 19 clinical isolates, distributed over several *C. albicans* phylogenetic groups (CdE, unpublished data). This suggests that they were most likely not responsible for the restoration of filamentation. Of the 120 remaining positions, 83 were in non-coding regions, 22 resulted in synonymous changes and 15 resulted in non-synonymous changes (S3 DS2-4 Table). Finally, the RNA-Seq dataset was used as an additional source to detect SNPs specifically in expressed genes (see Materials and Methods & S3 DS6&7 Table,): A total of 65 putative transcribed SNPs, both heterozygous and homozygous, were found in the Evo strain, of which 21 were located in non-coding regions. Inside ORFs, 26 caused a synonymous and 13 a non-synonymous nucleotide exchange. Of all 39 SNPs detected in coding regions, 24 were located in genes of the *ALS* gene family (*ALS2* and *ALS4*), although these are likely false positives, as genes of the *ALS* family possess a very high sequence similarity and tandem repeat regions complicating read-mapping and SNP resolution [49]. Comparison of SNPs detected by RNA-Seq and Whole-Genome Sequencing revealed three SNPs shared by both detection methods. One SNP was located in a non-coding region between two uncharacterized genes (orf19.351 and orf19.352), while the other two were located inside ORFs. A SNP in *ATP18* (orf19.2066.1) resulted in a synonymous amino acid exchange, while the second SNP in *SSN3* (orf19.794) resulted in a heterozygous, non-synonymous Arg/Arg to Arg/Gln amino acid change.

### A Mutation in *SSN3* is essential for the filamentous phenotype in the Evo strain

**Figure 6:**
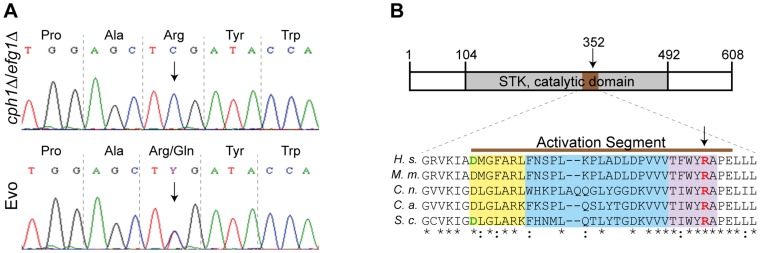
Single nucleotide polymorphism in *SSN3* of the Evo strain and location of the mutated amino acid. (**A**) Partial *SSN3* sequence for *cph1*Δ/*efg1*Δ and Evo strains flanking SNP 1055 (marked with an arrow). Notice the heterozygosity in the Evo strain. (**B**) Schematic view of the catalytic domain of Ssn3 (STK = serine/threonine kinase) with the position of the activation segment highlighted in brown and the amino acid exchange indicated by an arrow (top). Sequence alignment of the Ssn3 activation segment in different species (*H. s. Homo sapiens* [NP_001251.1], *M. m. Mus musculus* [NP_705827.2], *C. n. Cryptococcus neoformans* [XP_568416.1], *C. a. C. albicans* [XP_720918.1] and *S. c. Saccharomyces cerevisiae* [NP_015283.1]). The arrow indicates the amino acid exchange in the Evo strain. The Mg-binding loop is highlighted in yellow, the activation loop in blue and the P+1 loop in purple. Amino acids that are known to abrogate kinase activity when mutated are colored in green [51], [98]. Asterisks underneath the alignment indicate positions with conserved amino acids and colons indicate highly similar residues (bottom). The mutated arginine (red) is part of the highly conserved P+1 substrate recognition loop.

As the SNP at nucleotide position 1,055 in the *SSN3* ORF (Fig. 6A) was detected in both analyses, we focused our investigation on this specific mutation. Ssn3 has been well characterized in *Saccharomyces cerevisiae* as an RNA polymerase II holoenzyme-associated cyclin-dependent kinase of the Mediator complex contributing to transcriptional control [50]. It was shown that Ssn3 promotes the degradation of the transcription factor Ste12 by phosphorylation and thereby regulates *S. cerevisiae* filamentous growth [51]. As depicted in Fig. 6B, the heterozygous Arg^352^Gln mutation of Ssn3 in the Evo strain is located within the activation segment of the protein kinase catalytic domain. An amino acid sequence comparison of *C. albicans* Ssn3 to sequences from *S. cerevisiae*, *Cryptococcus neoformans*, *Mus musculus* and *Homo sapiens* demonstrated this arginine residue to be conserved from fungi to mammals. The activation segment comprises several conserved structural features: the magnesium binding loop, the activation loop and the P+1 loop, in which the mutation occurred. While the activation loop is the site of regulatory phosphorylation in many kinases, the P+1 loop forms a pocket that recognizes the substrate protein [52].

**Figure 7:**
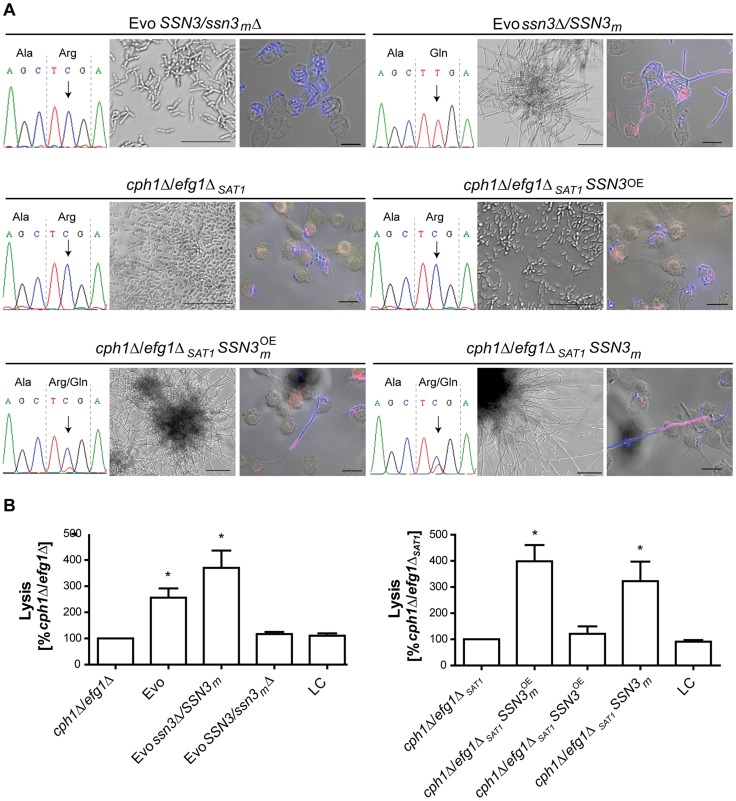
A single nucleotide polymorphism in *SSN3* is essential for filamentation. (**A**) Distinct impact on morphology by: deleting either the mutated (*SSN3*/*ssn3_m_*Δ) or the wild type *SSN3* allele (*ssn3*Δ*/SSN3_m_*) in the Evo strain, by overexpressing either the wild type *SSN3* allele (*cph1*Δ/*efg1*Δ*_SAT1_SSN3* ^OE^) or the mutated *SSN3* allele (*cph1*Δ/*efg1*Δ*_SAT1_SSN3*_m_^OE^) or by expressing the mutated *SSN3* allele from its native locus (*cph1*Δ/*efg1*Δ*_SAT1_SSN3*_m_) in a newly generated *cph1*Δ/*efg1*Δ*_SAT1_* strain. The partial *SSN3* sequences demonstrate the homozygosity or heterozygosity of the *SSN3* allele (left). Filamentous growth is visible in the Evo *ssn3*Δ/*SSN3_m_*, *cph1*Δ/*efg1*Δ*_SAT1_SSN3*_m_^OE^ and *cph1*Δ/*efg1*Δ*_SAT1_SSN3*_m_ strains after growth for 18 h at 37°C and 5% CO_2_ in DMEM+10% FBS and during co-incubation with macrophages, but not with the Evo *SSN3*/*ssn3_m_*Δ strain, *cph1*Δ/*efg1*Δ*_SAT1_* and *cph1*Δ/*efg1*Δ*_SAT1_SSN3* ^OE^ strains (scale bars: 18 h, 50 µm and MΦ, 20 µm; representative pictures are shown) (right). (**B**) Cell damage of macrophages caused by the different strains, as determined by lactate dehydrogenase (LDH) assay after 32 h of co-incubation. Robust host cell damage depends on the presence of the mutated allele *SSN3m*. Mean and SD of n = 4 (*p<0.05; compared to *cph1*Δ/*efg1*Δ and *cph1*Δ/*efg1*Δ*_SAT1_* respectively; LC = low control, medium only).

To ascertain the impact of the SNP on filamentation induction, we selectively deleted either the mutated or the wild type *SSN3* allele in the Evo strain, using the dominant selection marker *SAT1* [53]. Sanger sequencing confirmed the exclusive presence of either one allele in the genome (Fig. 7A). Strikingly, when incubated in DMEM with 10% serum at 37°C and 5% CO_2_ only the strain with the mutated allele still present (Evo *ssn3*Δ/*SSN3_m_*) was able to induce and maintain filamentation. The mutant containing only the wild type allele (Evo *SSN3*/*ssn3_m_*Δ) remained in the elongated yeast form, and thus presented the typical ancestral (*cph1*Δ/*efg1*Δ) phenotype (Fig. 7A). In addition, only the Evo *ssn3*Δ*/SSN3_m_* strain could escape from macrophages by forming filaments like the wild type (Fig. 7A). The damage capacity correlated with this ability to produce filaments: While Evo and Evo *ssn3*Δ*/SSN3_m_* strains showed the same levels of phagocyte lysis, the Evo *SSN3*/*ssn3_m_*Δ strain caused significantly less damage during co-incubation with macrophages. In fact, damage was indistinguishable from the original *cph1*Δ/*efg1*Δ strain (Fig. 7B). In contrast, the deletion of the mutated allele had no influence on the hyphal development defect on solid medium (S5A Figure) and sensitivity to cell wall disturbing agents (S5B Figure).

To further ascertain that the *SSN3* mutation alone is sufficient to allow filamentation in a *cph1*Δ/*efg1*Δ background, we created an independent *cph1*Δ/*efg1*Δ double mutant using the dominant selection marker *SAT1* (see S3 DS1 Table). Importantly, this *cph1*Δ/*efg1*Δ_SAT1_ strain contained neither the *URA3* amplification nor the trisomy of chromosome 7 or other genetic alterations of the original *cph1*Δ/*efg1*Δ strain. In all our filamentation assays, this mutant behaved identical to the original *cph1*Δ/*efg1*Δ strain by not forming any hyphae (Fig. 7A) and hence not escaping from or damaging macrophages (Figs. 7A & 7B). To isolate the effect of the mutated *SSN3*, we followed several strategies with this new mutant: *SSN3* overexpression strains were created of both the wild type and mutated (*SSN3*_m_) allele under the control of the strong *ADH1* promoter (see S1 Protocol). Strikingly, only the mutated allele allowed hyphae formation under inducing conditions in the *cph1*Δ/*efg1*Δ_SAT1_ strain (Fig. 7A, lower left corner), even in the presence of the two native *SSN3* alleles. Similarly, macrophage lysis was increased in the *SSN3*_m_ overexpressing strain, but not under *SSN3* overexpression (Fig. 7B, right panel). Finally, we integrated the mutated *SSN3* (together with a *SAT1* cassette) into the *SSN3* locus of *cph1*Δ/*efg1*Δ_SAT1_, replacing one *SSN3* allele and essentially reproducing the heterozygous situation of the Evo strain. Again, this strain behaved virtually identical to the Evo strain, both in forming hyphae (Fig. 7A) and in damaging macrophages (Fig. 7B, right panel).

In summary, these data show that a non-synonymous mutation in *SSN3* that arose during our microevolution experiment is alone sufficient for regaining the ability to filament even in the absence of Efg1 and Cph1.

## Discussion

Previous experimental studies on the acquisition of antifungal drug resistance and on stress-induced chromosome rearrangements have elegantly demonstrated the adaptive potential of *C. albicans* [44], [54]. Here, we demonstrate – to our knowledge for the first time – that a complex trait such as the hyphal formation program of *C. albicans* can be subject to microevolution in the laboratory.

The yeast-to-hyphae transition is of crucial importance for full *C. albicans* pathogenicity, which is reflected by its complex regulation [14]. Multiple overlapping as well as separate signaling pathways are activated by various environmental signals to regulate hyphae formation. Wild type hyphae are an important contributor to the fungus' ability to escape from engulfing macrophages. In contrast, the *cph1*Δ/*efg1*Δ mutant strain cannot escape by filament formation, yet is able to replicate inside macrophages and to block phagosome maturation. Therefore, we expected that the mutant strain would survive in the phagosome, albeit with reduced fitness compared to the wild type. We monitored the phenotypic changes of the *cph1*Δ/*efg1*Δ strain co-passaged with macrophages for 42 passages. On a comparatively short evolutionary timescale our experiment resulted in a strain which not only regained the ability to filament, but also re-acquired other important characteristics, like a more wild type-like cell wall structure and increased virulence. We were able to show that a minimal sequence alteration accounts for the striking phenotypic reversal to wild type-like filamentation: a single missense mutation in *SSN3*. *SSN3* encodes a fungal protein kinase, which phosphorylates various regulators in *S. cerevisiae*. Our data shows that it can become important for bypassing the requirements of Efg1 and Cph1 for filamentation in *C. albicans*.

The in-depth characterization of the evolved strain revealed that the hyphal morphogenesis program can be induced by certain, but not all conditions which induce filamentation in the wild type strain. The fact that the Evo strain filaments in liquid, but not on solid media indicates an involvement of cAMP signaling and hence argues for a bypass of Efg1 functions rather than Cph1 [55]–[57]. This was further supported by three additional findings. First, the yeast-to-filament switch occurred in response to either serum, GlcNAc or CO_2_, stimuli all known to trigger the activation of PKA signaling [23], [58]–[60]. Second, filamentation was entirely blocked by the addition of the quorum-sensing molecule farnesol which represses both cAMP-PKA and MAPK signaling pathways [61], [62]. The full restoration of filamentation when cAMP was added supports the involvement of the cAMP-PKA pathway. Third, the repressor of hyphae formation, Nrg1 is normally downregulated by the cAMP-PKA pathway, except in the presence of farnesol [63]. Transcriptome analysis showed *NRG1* expression to be downregulated in the Evo strain, but not in the *cph1*Δ/*efg1*Δ mutant. As 70% of the upregulated genes in the Evo strain contain an Nrg1 binding site, these data emphasize the likely importance of Nrg1 levels on filamentation of the Evo strain.

Given that the *cph1*Δ/*efg1*Δ mutant is strongly reduced in virulence [22], the almost wild type-level virulence in the Evo strain in our murine model was striking. Examination of kidney sections revealed filament formation of the Evo strain *in vivo*. Compared to wild type filaments, these were shorter and resulted in less pronounced tissue invasion, which is likely associated with the lower overall virulence compared to the wild type.

Three factors are likely to have contributed to the increased virulence of the Evo strain in the absence of Efg1 and Cph1: First, its ability to escape from macrophages like the wild type; second, its adhesion to host cells which was significantly higher than the *cph1*Δ/*efg1*Δ strain; and third, the ability to form filaments upon contact with epithelial cells, which is a prerequisite for both active penetration into and induced endocytosis by host cells [64]. Wächtler *et al.* [17] showed that filamentation alone is insufficient to cause damage of host cells. We therefore compared the damage capacities of the *cph1*Δ/*efg1*Δ and the Evo strains. The Evo strain exhibited a significantly increased potential to damage both macrophages and epithelial cells compared to the double mutant. The adaptation to macrophages was accompanied by differences in additional traits, such as resistance to cell wall stresses. In the *cph1*Δ/*efg1*Δ strain, the higher sensitivity to cell wall disturbing agents, as well as the modified exposure of cell wall components, likely reflect an altered cell wall organization which was restored in the Evo strain. This is supported by findings from a recent study by Zavrel *et al.* [32] which showed that deletion of *EFG1* alone affects cell wall architecture. In our strains, these modifications of the cell wall seemed to be mediated by the kinases Mkc1 and Cek1. Previous analyses carried out in *cek1*Δ and *mkc1*Δ mutants already indicated their direct relationship to cell wall composition and integrity [35], [65], [66].

By analyzing the differences in gene expression acquired during co-culture passaging with macrophages, we found that all genes belonging to the core filamentation network [38] were upregulated in the Evo strain. This suggests that during filamentation the Evo strain transcriptionally utilizes the complete filamentation program. The transcription factors Tec1, Brg1, Ume6, Rim101, Hac1 and Efh1, which are known to be involved in regulation of filamentation [24], [67]–[71], were also upregulated in the Evo strain. Together with Nrg1, they likely orchestrate filament formation in the Evo strain. For *UME6*, it has been shown that its transcription is repressed by Nrg1-Tup1 and that ectopic Ume6 expression in *cph1*Δ/*efg1*Δ can rescue the filamentation defect under certain conditions [69].

For the maintenance of hyphal extension, both *UME6* and *EED1* are central [27], [31] and both showed an increased expression in the evolved strain. Thus, the mechanisms of hyphal extension seems similar between Evo and wild type cells [27]. Hence, the transcriptional conditions for initiation and maintenance of filamentation, which comprise the release of repression and the upregulation of positive regulators of filamentation, are met in the Evo strain. Furthermore, a considerable number of transcripts specifically up- and downregulated in the Evo strain are both *Candida*-specific and uncharacterized. It is feasible, therefore, that these uncharacterized transcripts assumed a novel role specifically during filament formation in the Evo strain. This is especially true as the morphological switch is one of the best-investigated characteristics in *C. albicans*, and genes involved in this process are generally well studied. However, differential regulation of genes not clearly linked to the yeast-to-hyphal switch, including these genes, but also *WOR1* and *NAT4* (both involved in the white-opaque switching) and *SST2* (involved in the mating response pathway), could have been caused by the mutated Ssn3 kinase (see below). Finally, it also should be noted that, even in the absence of filamentation, *cph1*Δ/*efg1*Δ was able to upregulate certain genes described as hyphae-associated under the condition tested here (incubation in DMEM+10% FBS at 37°C and 5% CO_2_ on a plastic surface). This is in disagreement with previous data showing that *EFG1* is required for expression of several hyphae-associated genes [23], [72]. It is possible, however, that alternative pathway(s), such as the Rim101 pH response pathway, are involved, as the cells were simultaneously exposed to diverse stimuli for filamentation. However, these genes still showed an increased induction in the Evo strain compared to the *cph1*Δ/*efg1*Δ strain, which argues for a further adaptation-induced, filament-associated change in regulation.

It has been demonstrated that acquired drug resistance in *C. albicans* is often accompanied by aneuploidy and/or isochromosome formation [54], [73] and that several stress conditions can enhance the rates of LOH events likely by mitotic recombination [44]. However, we did not detect any LOH events between the *cph1*Δ/*efg1*Δ and the Evo strain. The chromosome 7 trisomy was present initially in the *cph1*Δ/*efg1*Δ strain [74] and the Evo strain restored disomy by loss of one copy. The remaining gross genetic difference, an *URA3* amplification in the Evo strain can be explained by an insufficient Ura3 expression from the *EFG1* locus. An amplification of the gene may have increased fungal fitness during our experiment by ensuring more transcripts and hence more efficient growth. Prior studies suggested that ectopic expression of *URA3* influences the phenotypes of a diverse range of mutants [75], [76], and duplication of a *hisG*-*URA3*-*hisG* cassette resulted in restored filamentation of an *hwp1*Δ mutant [77]. We were able to exclude these Ura3 effects as causes for the Evo strain filamentation, as the acquired filamentation phenotype was maintained after removal of the multiple *URA3* copies. Furthermore, after re-introduction of a single copy of *URA3*, no differences in virulence traits, like adhesion or invasion, were detectable as compared to the multi-copy strains. Overall, these data and the fact that the observed filamentation and other phenotypes persisted even after repassaging in rich (YPD) medium, argued for small-scale genomic alterations, rather than epigenetic changes, acquired by *cph1*Δ/*efg1*Δ cells adapting to macrophages.

Comparative genome sequencing (by WGS and RNA-Seq) of *cph1*Δ/*efg1*Δ and Evo strains allowed us to pinpoint the microevolutionary changes in the Evo strain at the single nucleotide level. By combining the different approaches, we detected an expressed SNP in *SSN3*, which resulted in an Arg-to-Gln change at a highly conserved position within the presumable protein kinase domain. This SNP and thus gain of heterozygosity was found to be central for the yeast-to-filament transition of the Evo strain. Deletion of the mutated *SSN3* allele prevented the morphological switch in the Evo strain during growth under filament-inducing conditions and interaction with several types of host cells. Other phenotypes specific to the Evo strain were not affected by the deletion of the mutated *SSN3* allele, suggesting that they evolved independently from filamentation.

Importantly, introduction of a single mutated allele into an independent *efg1*Δ/*cph1*Δ strain fully copied the filamentation and host cell damage phenotype of the Evo strain. This strain contained neither the multiple *URA3* copies nor the trisomy of chromosome 7 or any other possible genetic alterations of the original *efg1*Δ/*cph1*Δ strain. Hence, the *SSN3* mutation alone bypassed the lack of the central transcription factors Cph1 and Efg1 and restored the ability to cause host cell damage *in vitro*, and likely to induce higher virulence *in vivo*.

Ssn3 itself (also referred as Srb10 or Cdk8) is part of the CDK (cyclin-dependent kinase) module (SRB10/11) of the Mediator complex, which is a regulator of RNA-polymerase II (RNAP II) activity [78], [79]. This CDK module phosphorylates the largest subunit of RNAP II, and Ssn3 additionally has roles in both transcriptional activation and repression in response to physiological signals, coordinating gene expression. By regulating the stability of the two important regulators, Ste12 (ortholog of Cph1) and Phd1, Ssn3 in *S. cerevisiae* is involved in the differentiation of yeasts into pseudohyphae under nutrient-limiting conditions [51], [80]. Interestingly, a kinase-deficient Asp290Ala Ssn3 only weakly phosphorylates Ste12 *in vitro*, and the lack of phosphorylation increases its stability [51]. Moreover, the catalytic activity of Ssn3 contributes to the repression of a subset of Tup1-regulated genes [81]–[83] in *S. cerevisiae*. Tup1 is recruited to promoters by Nrg1 [84], a factor which was downregulated in the Evo strain.

Although the precise signaling pathway(s) controlling Ssn3 remain to be determined, Chang *et al.* [85] showed that the activity of Srb9, another subunit of the CDK kinase module, is regulated by the PKA signaling pathway in *S. cerevisiae*. Based on our data it is tempting to speculate that the activation of the cAMP-PKA pathway results in activation of Ssn3 kinase activity, and the observed filament-specific transcriptional changes may thus depend on either a reduced or absent substrate recognition or on impaired substrate phosphorylation activity due to the Arg^352^Gln substitution. It is supposable that a loss of the substrate-specific kinase activity increases the stability of positive regulator(s) of filamentation by reducing their phosphorylation. Alternatively (or in addition), the impaired kinase activity could lead to a derepression of genes associated with positive regulation of filamentous growth. Importantly, in this model the kinase-deficient Ssn3 remains part of the Mediator complex, and could fulfill any additional function it may have (e.g. in the structure or recruitment of additional proteins). In both models, a decreased kinase activity would reduce inhibitory effects on filamentation, and hence would increase the sensitivity of the filamentation network to external stimuli. This would likely allow to bypass the need for additional Efg1 signaling. Additional genes outside of the immediate filamentation network may also be affected, as this model implies a pleiotropic effect of the Ssn3 mutation, with several transcription factors as possible clients.

Thus, it seems that not the disrupted cAMP-PKA signaling pathway itself evolved in our microevolution experiment, but instead a regulatory hub for filamentation which the pathway probably targets in addition to Efg1. In this hub, even single or few mutations seem to be able to lead to striking phenotypic alterations, as many filament-associated genes are directly or indirectly targeted. Finally, it is interesting to speculate why only one *SSN3* allele was mutated, and we did not observe any LOH event to homozygosity at this locus. It seems possible that one mutated allele alone was sufficient to promote filamentation in macrophages, while the other wild type allele, still capable of full phosphorylation activity, was still required for additional functions of Ssn3. This is somewhat supported by the observation that overexpression of the mutated *SSN3* allele in a background with the native *SSN3* alleles still in place was sufficient to allow hyphae formation. In our model, the mutated Ssn3 competes with the wild type Ssn3, and overexpression allows the mutated protein to gain entry into a sufficient number of Mediator complexes.

In conclusion, using the nonfilamentous mutant *cph1*Δ/*efg1*Δ, we have shown that *C. albicans* can rescue one of its key virulence traits, the yeast-to-hyphal switch, with a single nucleotide change when put under adequate selection pressure. A mutation in the transcriptional regulator Ssn3 adaptively rewired the transcription network to enable filamentation in response to external cues while bypassing the need for Efg1 and Cph1. This shows an unexpected robustness of the whole filamentation system even to severe disruptions, and a high degree of adaptability. The selection scenario we used - co-incubation with macrophages - clearly reflects a condition *C. albicans* encounters in the host and thus might be an evolutionary pressure that can shape the infection biology of this fungus. In fact, this hypothesis is supported by another evolution experiment, which analyzed the adaptation of *C. glabrata* to macrophages. There, the selection pressure resulted in the appearance of a strain with pseudohyphae-like structures and increased virulence again by a single nucleotide mutation [86]. This demonstrates that during interaction with the host or host cells, significant changes in morphology and virulence are possible on a very short evolutionary time-scale.

## Materials and Methods

### Ethic statement

All animal experiments were in compliance with the German animal protection law and were approved by the responsible Federal State authority (Thüringer Landesamt für Lebensmittelsicherheit und Verbraucherschutz) and ethics committee (beratende Kommission nach § 15 Abs. 1 Tierschutzgesetz; permit no. 03-007/07).

Body surface temperature and body weight were recorded daily and animals were monitored twice a day for disease progression. Mice showing severe signs of illness (isolation from the group, apathy, hypothermia and drastic weight loss) were humanely sacrificed by ketamine/xylazine overdose and exsanguination.

### Strains and growth conditions

*Candida albicans* strains and mutants used in this study are listed in S1 Table. Strains were grown in YPD medium (1% peptone, 1% yeast extract, 2% glucose and optionally 2% agar) or SD medium (2% dextrose, 0.17% yeast nitrogen base, 0.5% ammonium sulfate and optionally 2% agar) at 30°C. Uridine (50 µg/ml) or nourseothricin (NAT; 100 µg/ml) were added as required. If not stated otherwise, stationary phase cells were used in the experiments. Mutants were constructed as described in Protocol S1.

### Cell lines

The murine peritoneal macrophage-like cell line J774A.1 (DSMZ) and the human buccal carcinoma epithelial cell line TR-146 (Cancer Research Technology) were grown in Dulbecco's Modified Eagle's Medium (DMEM, PAA) supplemented with 10% FBS (PAA) and routinely cultured until passage 20. Both cell lines were maintained at 37°C under 5% CO_2_. J774A.1 cells were removed from tissue-culture flasks by gentle scraping, while TR-146 cells were enzymatically harvested by Accutase (PAA) treatment.

### Evolution experiment

About 8×10^6^ J774A.1 macrophages were seeded into a 75 cm^2^ cell culture flask with DMEM supplemented with 10% FBS and 1% Penicillin/Streptomycin (PAA). For the evolution experiment, macrophages were initially infected with 4×10^6^ cells of the *cph1*Δ/*efg1*Δ strain. After that, 4×10^6^ re-isolated *C. albicans* cells were transferred to a fresh macrophage culture. After 24 h of co-incubation, infected macrophages were washed (3× with PBS) and lysed with 2 ml lysis buffer (50 mM Tris, 5 mM EDTA, 150 mM NaCl and 0.5% Nonidet P40 [Sigma-Aldrich]). The lysate was transferred to a 2 ml reaction tube and fungal cells were collected by centrifugation. The *C. albicans* cells were washed two times with DMEM and counted before infection of fresh macrophages.

To verify the absence of *EFG1* and *CPH1*, Southern blot analysis was performed for the Evo strain as described previously [22]. Briefly, genomic DNA (gDNA) was digested with *Ava*II or *Kpn*I to verify *EFG1* or *CPH1* deletion, respectively. DIG-labeled probes were generated (Roche) using genomic DNA from the strain SC5314 and primers EFG-A/EFG-B and P33/CPH-B (S1 Table).

### Phenotypic characterization

A detailed description of the phenotypic analyses can be found in Protocol S1.

### Staining procedures and detection of β-1,3-glucans and mannans

Fungal cells were grown in DMEM+10% FBS on glass coverslips in a 24 well microtiter plate for filipin (Sigma), calcofluor white (CFW) and Als3 immunostaining. Flow cytometry was used to quantify mannan and β-1,3-glucan exposure on the surface of stationary *C. albicans* cells after staining with concanavalin A and anti- β-1,3-glucan. Piercing and invasion rate were determined by differential staining. All staining procedures are described in Protocol S1. Epifluorescence (Leica DM5500B, Leica DFC360 FX) was used to detect CFW and filipin (DAPI filter), Alexa Fluor 488 (FITC filter) and Alexa Fluor 647 (Cy5 filter). Micrographs were taken with a Leica Digital Camera DFC360 FX or a Zeiss AxiCam ICc3.

### Replication and piercing assay

Two times 10^5^ J774A.1 macrophages were seeded onto glass cover slips placed in 24 well microtiter plates and allowed to adhere overnight. Non-adherent macrophages were removed by washing with PBS. To monitor intracellular replication, *C. albicans* cells were labeled with 100 µg/ml fluorescein isothiocyanate (FITC, Sigma-Aldrich) in carbonate buffer (0.1 M Na_2_CO_3_, 0.15 M NaCl, pH 9.0) for 30 min at 37°C and washed 3× with PBS. To quantify piercing rates, cells were washed without prior staining. Two times 10^5^ fungal cells were added to macrophages in DMEM+10% FBS. The plates were incubated for indicated timepoints (see figure legends). Cells were then washed once with PBS and fixed with 4% paraformaldehyde. Intracellular replication was detected by fluorescence microscopy after mounting the samples in ProLong Gold Antifade Reagent with DAPI (Invitrogen). After co-incubation, piercing of macrophages by filaments was quantified by differential staining. The assays were performed in biological triplicates.

### Adherence and invasion assay

Two times 10^5^ TR-146 epithelial cells were seeded onto glass cover slips placed in 24 well microtiter plates and cultured for 2–3 days to 95%-100% confluency. Adherence and invasion assays were performed as previously described [17]. Briefly, to determine the adhesion rate, TR-146 monolayers were infected with 1×10^6^ *C. albicans* cells. After one hour of co-incubation, non-adherent yeast cells were removed by rinsing 3× with PBS. Cells were fixed with 4% paraformaldehyde, permeabilized with 0.5% Triton X-100 and adherent *C. albicans* cells were stained with CFW for fluorescence microscopy. Invasion rates were determined by infecting TR-146 monolayers with 1×10^5^ *C. albicans* cells. After incubation, cells were fixed and differentially stained for fluorescence microscopy. Both assays were repeated at least three times.

### Quantification of damage to host cells

Five times 10^4^ host cells (J774A.1 or TR-146) were seeded in 96 well microtiter plates. J774A.1 macrophages were cultured for 1 day before use, while TR-146 epithelial cells were cultured for 2 days to 95%–100% confluency. Damage of macrophages and epithelial cells was determined by measuring the release of lactate dehydrogenase (LDH) with the Cytotoxicity Detection Kit (Roche Applied Science) following 32 h of co-incubation with 5×10^4^ *C. albicans* cells according to the manufacturer's protocol. The experiments were performed as previously described [17] and repeated at least three times.

### Murine infection model

For survival studies the intravenous challenge model for disseminated *C. albicans* infection was used. Six to eight weeks old female BALB/c mice (18–20 g) purchased from Charles River were used for the experiments. Mice were challenged intravenously with 5×10^5^ *C. albicans* cells in 200 µl PBS via the lateral tail vein. All mice surviving to day 20 were humanely sacrificed. For histology, kidneys were collected and fixed with buffered formalin and paraffin-embedded sections were stained with Periodic acid-Schiff (PAS) according to standard protocols.

### Western blot analysis

To detect phosphorylated Mkc1 and Cek1 as well as α-tubulin, cells of an overnight culture were adjusted to an OD_600_ of 0.5 in SD medium (control) or SD medium supplemented with 450 µg/ml congo red, and incubated for 4 hours at 30°C. Cell disruption, protein extraction and western blot analysis using anti-phospho-p44/42 MAP kinase antibody (Cell Signalling Technology) and rat anti-α-tubulin antibody (AbD Serotec), respectively, were performed as previously described [87].

### RNA sample preparation and isolation

*C. albicans* cells from an overnight culture were diluted to OD_600_ = 0.2 in YPD medium and grown to log-phase for 4 h at 30°C. Cells were collected by centrifugation and a zero time point sample was frozen in liquid nitrogen until RNA extraction. In addition, 1×10^7^ cells were incubated one hour under filament-inducing conditions (DMEM+10% FBS at 37°C and 5% CO_2_ in a 75 cm^2^ cell culture flask). For farnesol experiments, 10 µM farnesol was added to the medium just prior to the experiment. After incubation, medium and non-adherent cells were removed and 5 ml ice-cold PBS was added. The cells were collected by scraping and then centrifuged for 5 min at 6,000 g at 4°C. Cell pellets were snap frozen in liquid nitrogen. Total RNA was isolated using the Ribopure-Yeast Kit (Ambion) and treated with Turbo DNase (Ambion). RNA quality was determined in a Bioanalyzer with an RNA 6000 Nano LabChip Kit (Agilent Technologies) according to the manufacturer's protocol. RNA concentration was determined with a Nanodrop ND1000 (Peqlab).

### Copy number determination and quantitative gene expression analysis

Copy number and expression levels of selected genes were analyzed with a my-Budget 5× EvaGreen QPCR Mix II (Bio&Sell) in a C1000TM Thermal Cycler (BioRad) using gene-specific primers (S1 Table). For expression analysis, 600 ng of total RNA was reversely transcribed with the SuperScript III First-Strand Synthesis Kit (Invitrogen) according to the manufacturer's instructions. *URA3* gene copy number was determined from 100 ng of gDNA with primers URA3-fw and URA3-re (S1 Table). PCR conditions were as followed: 95°C for 15 min, 40 cycles of each 95°C for 15 s, 60°C for 40 s and 72°C for 15 s. A melting profile was generated to confirm PCR product specificity. Relative gene expression levels were determined by the 2^ΔΔCt^ method [88] with *ACT1*, *EFB1* and *PMA1* as internal controls. *URA3* copy number was calculated with *ACT1* internal control and gDNA from SC5314 (containing two copies of *URA3*) as reference. Three independent experiments were performed.

### Pulsed-field gel electrophoresis (PFGE) and SNP-RFLP analysis

PFGE and SNP-RFLP are described in Protocol S1.

### RNA sequencing, transcriptional profiling and SNP discovery from RNA-Seq data

In order to use only high quality reads, trimming was performed using Btrim (window size = 15, average quality score = 20) [89]. For differential gene expression analysis high quality trimmed reads were mapped against the sequence Assembly 21 of strain SC5314 [48] using the spliced read mapper TopHat 2.0.6 [90] with the “known transcripts” (-G option) and uniquely mapped reads were counted using HTSeq [86]. Raw counts for each gene were loaded into R and differentially expressed genes were identified using the packages edgeR and DESeq [91], [92] and filtered by adjusted p-values (<0.01) and RPKM value (≥1). Data were deposited at the Gene Expression Omnibus (GSE56174) and can be found in S2 Table. Nrg1 binding sites (A/C)(A/C/G)C_3_T in putative promoter regions (usually −1000 bp/+50 bp) of all *C. albicans* genes were determined by SiTaR [93] allowing no mismatch. Fisher's exact test was used to determine if the Nrg1 motif-containing promoters were overrepresented in genes specifically upregulated twofold in the Evo strain, as compared to all remaining genes. For SNP calling quality trimmed reads from all samples of each strain were merged and the protocol of GATK [94] with slight changes was followed (i.e. reads were mapped using BWA algorithm [95], duplicates were removed and realignment around indels and base recalibration was performed). Next, we used bam-readcount (www.github.com/genome/bam-readcount), which determines the nucleotide distribution at each single base. Heterozygous SNPs were defined as positions where 25% or more of the reads showed an alternative nucleotide. Homozygous SNPs were defined as positions where more than 90% of the reads differed from the reference. Minimum nucleotide sequence depth was 20. Clustal Omega [96] was used for multiple sequence alignments.

### Whole genome sequencing and sequence analysis

Genomic DNA isolated from the *cph1*Δ/*efg1*Δ and Evo strains were processed to prepare libraries for Illumina sequencing, and the TruSeq DNA Sample Prep kit (Illumina) was used according to the manufacturer's recommendations. DNAs were randomly fragmented by sonication to an average fragment length of 500 bp and Illumina adapters were blunt-end ligated to the fragments. The final libraries were amplified by PCR followed by sequencing on an Illumina Genome Analyzer platform (Illumina GAII). 60 nt single-end reads were aligned to the *C. albicans* strain SC5314 reference genome [48] downloaded on 02/24/2012 using shore 5.0 [97]. Sequencing depth scores were computed for each 1 kb region across the genomes and for ORFs using sequencing depth data for each nucleotide located within the 1 kb region or the ORF. Sequencing depth scores were normalized based on the overall sequencing depth obtained for each genome. Single nucleotide polymorphisms were identified using shore 5.0 [97] at positions covered at least 30 times with a minimum quality of 25. Homozygous SNPs were defined as positions where 90% of the reads meeting these criteria differed from the reference genome. Heterozygous SNPs were defined as positions where 20% or more of the reads showed one allele and 80% or less of the reads showed a second allele.

### Statistical analysis

Data were visualized and statistically analyzed using GraphPad Prism version 5.00 (GraphPad Software, USA). Statistical analyses were performed by 1-way ANOVA (mannan and β-1,3-glucan exposure) or 2-way ANOVA (piercing, adhesion, invasion, damage and gene expression) followed by a Bonferroni correction. Differences in survival of mice were evaluated by Log-rank (Mantel-Cox) test.

## Supporting Information

S1 Figure Screening of *C. albicans* deletion mutants for defects in hyphal formation during interaction with macrophages and verification of the *cph1*Δ/*efg1*Δ genotype in the Evo strain. (**A**) Morphologies of different *C. albicans* mutants and the corresponding wild type strains upon phagocytosis by macrophages (J774A.1). Note that only the double mutant strain cannot escape from macrophages. Figures show overlay of DIC and fluorescent images. *C. albicans* appears blue (CFW) and extracellular section of hypha red (ConA). Arrows highlight piercing of macrophage membrane by *C. albicans* cells (scale bar: 10 µm). (**B**) The WT (CAI4+CIp10) forms hyphae after phagocytosis (white arrows), while the *cph1*Δ/*efg1*Δ strain replicates intracellularly in J774A.1 cells (black arrows). Yeast cells were stained with FITC prior to infection. Six hours after infection samples were fixed and stained with DAPI for analysis by fluorescence microscopy. FITC is not transferred to hypha or new daughter cells during cell division (scale bar: 10 µm, representative picture). (**C**) Southern blots of wild type (WT), *cph1*Δ/*efg1*Δ and Evo strains confirm the deletion of *EFG1* (left) and *CPH1* (right) in the Evo strain. Genomic DNAs were digested with either *Ava*II or *Kpn*I, and DNA molecular weight marker III, DIG labeled (Roche) was used as size standard.
(TIF)

S2 Figure Morphological index and morphology of the Evo strain under different conditions. (**A**) Overnight cultures of *cph1*Δ/*efg1*Δ and Evo strains were diluted into DMEM+10% FBS and incubated for the indicated time points at 37°C and 5% CO_2_ on cover slips. After fixation the percentage of yeast, pseudohyphal and hyphal cells was quantified using the morphological index (MI) [26]. Mean+SD of at least 100 cells in two experiments. (**B**) Morphology of *cph1*Δ/*efg1*Δ and Evo strains in phase contrast under different filament-inducing conditions. The Evo strain formed filaments in response to stimuli other than used during the evolution experiment. (**C**) Colony morphology of strains under embedded conditions. The Evo strain exceeds the hyperfilamentation phenotype of the *cph1*Δ/*efg1*Δ strain. (**D**) Colony morphology of analyzed strains grown on solid YPD agar supplemented with 10% FBS for 6 days, on solid Spider and Lee's medium for 9 days, and on solid YNB agar supplemented with 2% glucose and 10 mM urea for 11 days at 37°C (scale bar: 1 mm; representative pictures are shown). Only WT forms filaments under these conditions.
(TIF)

S3 Figure Results of the RNA-Seq analysis. (**A**) RNA-Seq results are in good agreement with qRT-PCR analyses of four selected genes. Expression was normalized against three housekeeping genes (*ACT1*, *EFB1* and *PMA1*). Fold change gene expression of filament-inducing condition versus yeast promoting condition (YPD, 30°C) is shown (mean+SD). (**B**) Venn diagrams of differentially expressed genes of *cph1*Δ/*efg1*Δ and Evo strains during growth in DMEM+10% FBS at 37°C and 5% CO_2_ on a plastic surface compared to yeast promoting condition (YPD, 30°C). (**C**) Expression heat map of the eight genes of the core filamentation response in the *cph1*Δ/*efg1*Δ and Evo strains during growth in DMEM+10% FBS at 37°C and 5% CO_2_ with white boxes indicating no differential expression (NDE).
(TIF)

S4 Figure Analysis of the genome architecture of the Evo strain. (**A**) Whole genome profiles of wild type (WT), *cph1*Δ/*efg1*Δ and Evo chromosomes separated by PFGE and stained with ethidium bromide are identical among all strains analyzed. (**B**) Whole-genome sequencing of *cph1*Δ/*efg1*Δ and Evo strains revealed a chromosome 7 (Chr 7) trisomy in the *cph1*Δ/*efg1*Δ strain (top) and an amplification of *URA3* on chromosome 3 (Chr 3) in the Evo strain (bottom). Normalized log_2_ read depth per 1 kb region along the chromosomes are shown. (**C**) Relative expression from the RNA-Seq experiment between *cph1*Δ/*efg1*Δ and Evo. The chromosome 7 trisomy of *cph1*Δ/*efg1*Δ is reflected by a mean 1.5× higher expression of genes specifically on this chromosome. Other chromosomes showed no change in mean expression (shown here for chromosome 6 as an example) (**D**) Verification of *URA3* amplification by qPCR analysis using genomic DNA isolated from the *cph1*Δ/*efg1*Δ and Evo strains. Copy number of *URA3* was normalized to *ACT1*. Mean+SD. (**E**) The *URA3* gene was successfully deleted from the Evo Ura^−^ strain. Whole chromosomes of the Evo strain and 5-fluoroorotic acid (FOA) treated Evo cells (Evo Ura^−^) were separated by PFGE, stained with ethidium bromide (left) and subjected to Southern hybridization with a *URA3* probe to verify successful deletion of all *URA3* copies in the genome (right). (**F**) Phenotype of Evo and Evo Ura^−^ strains after growth for 18 h at 37°C and 5% CO_2_ in DMEM+10% FBS (representative pictures). Removal of all *URA3* copies has no influence on the regained filamentation properties of the Evo strain. (**G**) Virulence traits of the *efg1*Δ/*cph1*Δ (ΔΔ) and Evo strains cured of *URA3* (Ura-) and after subsequent re-introduction of a single *URA3* copy using the CIp10 plasmid for genomic integration. No difference can be detected between the multi-copy and single-copy strains in adhesion or invasion of epithelial cells, or damage to macrophages. Absence of *URA3* reduces macrophage damage, probably due to the low uridine concentration inside the phagosome.
(TIF)

S5 Figure Characterization of the Evo *SSN3*/*ssn3_m_*Δ and Evo *ssn3*Δ*/SSN3_m_* strains. (**A**) Colony morphology of the Evo *ssn3*Δ/*SSN3_m_* strain grown on solid Lee's medium, Spider medium and YPD medium supplemented with 10% FBS for 6 days at 37°C (scale bar: 1 mm; representative pictures are shown). Observed morphologies resemble those of the Evo strain. (**B**) Stress resistance of *cph1*Δ/*efg1*Δ, Evo, Evo *SSN3*/*ssn3_m_*Δ and Evo *ssn3*Δ*/SSN3_m_* strains against different cell wall perturbing agents. The presence of either *SSN3* allele has no influence on the increased stress resistance of the Evo strain. Experiments yielded similar results for at least three replicates.
(TIF)

S1 Table Strains and primers used in the study.
(DOC)

S2 Table Excel data sheets contain data for differentially expressed genes in the *cph1*Δ/*efg1*Δ and the Evo strains during incubation under filament-inducing condition (DMEM+10% FBS at 37°C and 5% CO_2_ on a plastic surface) identified by RNA-Seq analysis (log_2_ RPKM data and raw read counts for all annotated transcripts and novel transcripts identified by Bruno *et al.* [37]).
(XLS)

S3 Table SNP-RFLP analysis of 32 SNP-RFLP markers (two per chromosome arm) to detect large-scale changes in the genomes of the *cph1*Δ/*efg1*Δ and Evo strains and predicted SNPs discovered by whole-genome sequencing and RNA-Seq of *cph1*Δ/*efg1*Δ and Evo strains.
(XLS)

S1 Protocol Additional methods used in this manuscript and for generation of the supplementary figures.
(DOC)
