# Supplementary figures and images for: Microevolution of Candida albicans in Macrophages Restores Filamentation in a Nonfilamentous Mutant
Source: PLoS Genet. 2014 Dec 4;10(12):e1004824. doi: 10.1371/journal.pgen.1004824 (PMC4256171; doi:10.1371/journal.pgen.1004824)

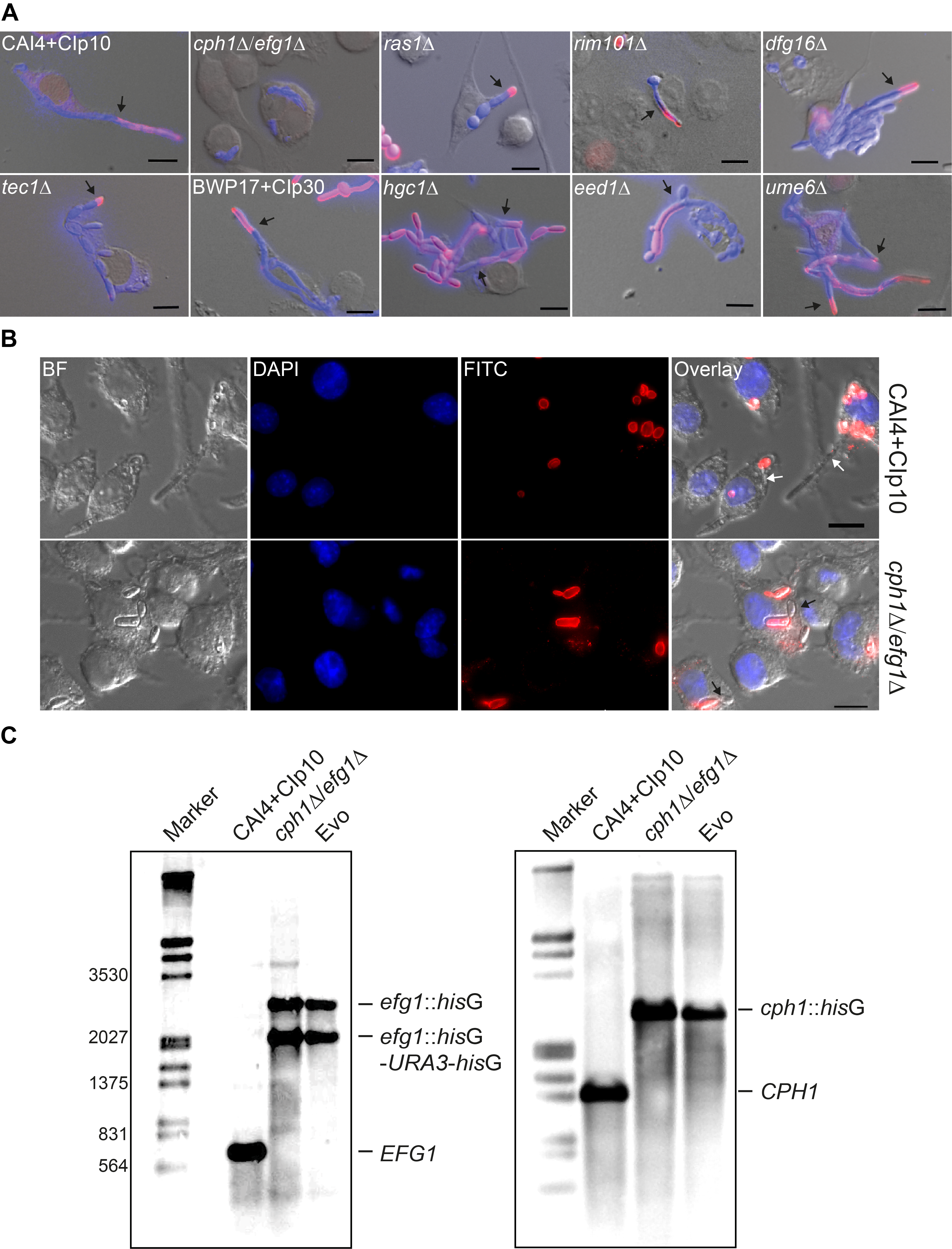

Supplement: S1 Figure — Screening of C. albicans deletion mutants for defects in hyphal formation during interaction with macrophages and verification of the cph1Δ/efg1Δ genotype in the Evo strain. (A) Morphologies of different C. albicans mutants and the corresponding wild type strains upon phagocytosis by macrophages (J774A.1). Note that only the double mutant strain cannot escape from macrophages. Figures show overlay of DIC and fluorescent images. C. albicans appears blue (CFW) and extracellular section of hypha red (ConA). Arrows highlight piercing of macrophage membrane by C. albicans cells (scale bar: 10 µm). (B) The WT (CAI4+CIp10) forms hyphae after phagocytosis (white arrows), while the cph1Δ/efg1Δ strain replicates intracellularly in J774A.1 cells (black arrows). Yeast cells were stained with FITC prior to infection. Six hours after infection samples were fixed and stained with DAPI for analysis by fluorescence microscopy. FITC is not transferred to hypha or new daughter cells during cell division (scale bar: 10 µm, representative picture). (C) Southern blots of wild type (WT), cph1Δ/efg1Δ and Evo strains confirm the deletion of EFG1 (left) and CPH1 (right) in the Evo strain. Genomic DNAs were digested with either AvaII or KpnI, and DNA molecular weight marker III, DIG labeled (Roche) was used as size standard. (TIF) [file pgen.1004824.s001.tif]

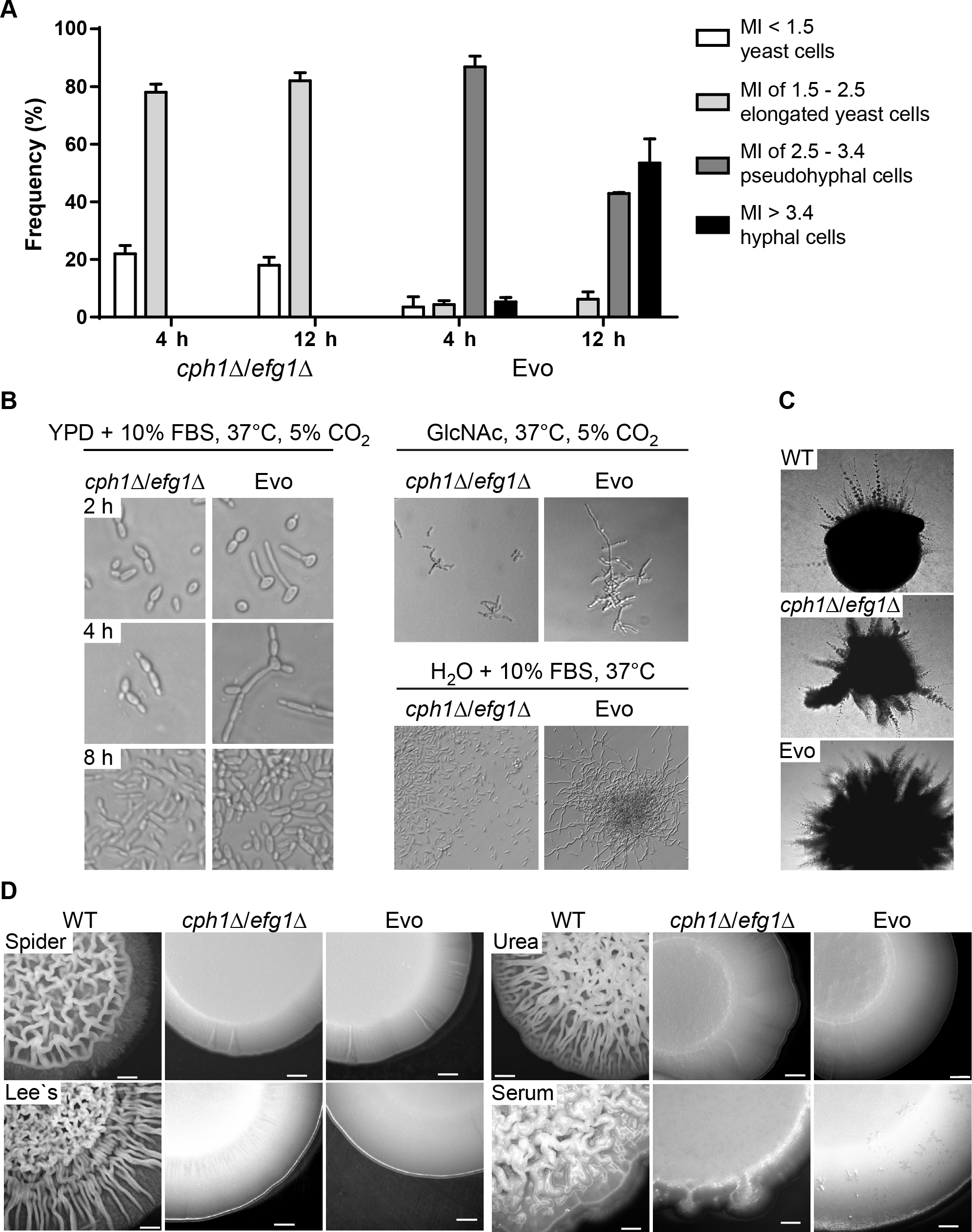

Supplement: S2 Figure — Morphological index and morphology of the Evo strain under different conditions. (A) Overnight cultures of cph1Δ/efg1Δ and Evo strains were diluted into DMEM+10% FBS and incubated for the indicated time points at 37°C and 5% CO2 on cover slips. After fixation the percentage of yeast, pseudohyphal and hyphal cells was quantified using the morphological index (MI) [26]. Mean+SD of at least 100 cells in two experiments. (B) Morphology of cph1Δ/efg1Δ and Evo strains in phase contrast under different filament-inducing conditions. The Evo strain formed filaments in response to stimuli other than used during the evolution experiment. (C) Colony morphology of strains under embedded conditions. The Evo strain exceeds the hyperfilamentation phenotype of the cph1Δ/efg1Δ strain. (D) Colony morphology of analyzed strains grown on solid YPD agar supplemented with 10% FBS for 6 days, on solid Spider and Lee's medium for 9 days, and on solid YNB agar supplemented with 2% glucose and 10 mM urea for 11 days at 37°C (scale bar: 1 mm; representative pictures are shown). Only WT forms filaments under these conditions. (TIF) [file pgen.1004824.s002.tif]

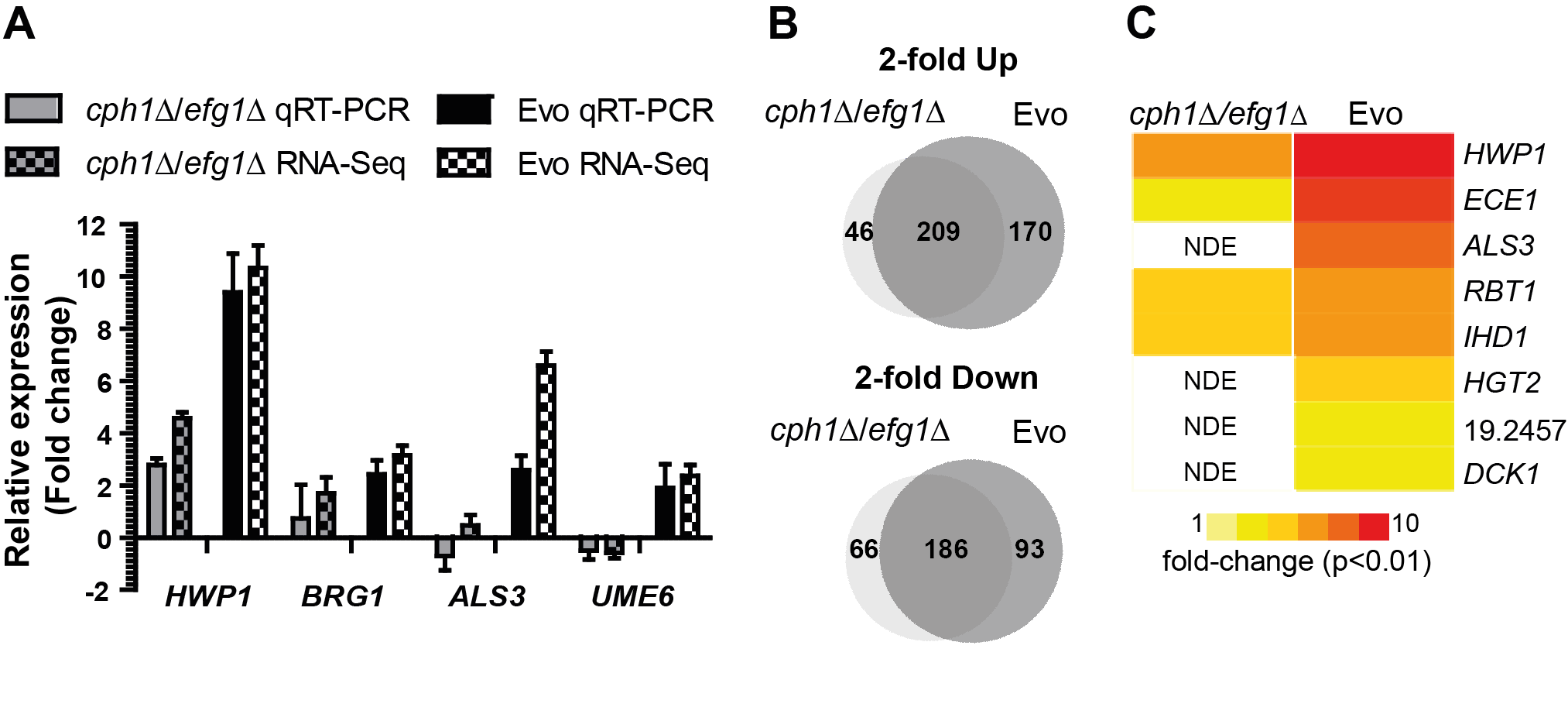

Supplement: S3 Figure — Results of the RNA-Seq analysis. (A) RNA-Seq results are in good agreement with qRT-PCR analyses of four selected genes. Expression was normalized against three housekeeping genes (ACT1, EFB1 and PMA1). Fold change gene expression of filament-inducing condition versus yeast promoting condition (YPD, 30°C) is shown (mean+SD). (B) Venn diagrams of differentially expressed genes of cph1Δ/efg1Δ and Evo strains during growth in DMEM+10% FBS at 37°C and 5% CO2 on a plastic surface compared to yeast promoting condition (YPD, 30°C). (C) Expression heat map of the eight genes of the core filamentation response in the cph1Δ/efg1Δ and Evo strains during growth in DMEM+10% FBS at 37°C and 5% CO2 with white boxes indicating no differential expression (NDE). (TIF) [file pgen.1004824.s003.tif]

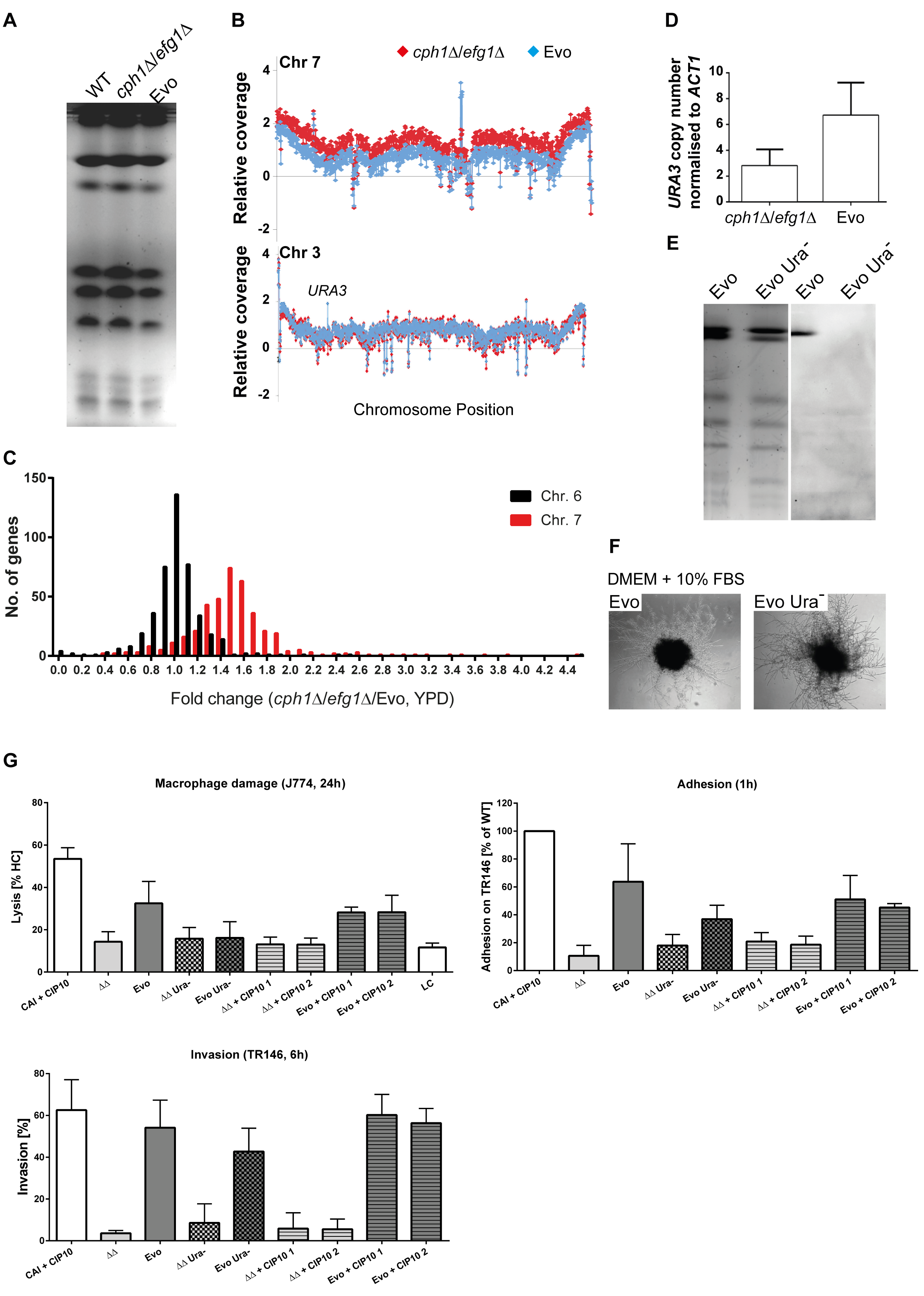

Supplement: S4 Figure — Analysis of the genome architecture of the Evo strain. (A) Whole genome profiles of wild type (WT), cph1Δ/efg1Δ and Evo chromosomes separated by PFGE and stained with ethidium bromide are identical among all strains analyzed. (B) Whole-genome sequencing of cph1Δ/efg1Δ and Evo strains revealed a chromosome 7 (Chr 7) trisomy in the cph1Δ/efg1Δ strain (top) and an amplification of URA3 on chromosome 3 (Chr 3) in the Evo strain (bottom). Normalized log2 read depth per 1 kb region along the chromosomes are shown. (C) Relative expression from the RNA-Seq experiment between cph1Δ/efg1Δ and Evo. The chromosome 7 trisomy of cph1Δ/efg1Δ is reflected by a mean 1.5× higher expression of genes specifically on this chromosome. Other chromosomes showed no change in mean expression (shown here for chromosome 6 as an example) (D) Verification of URA3 amplification by qPCR analysis using genomic DNA isolated from the cph1Δ/efg1Δ and Evo strains. Copy number of URA3 was normalized to ACT1. Mean+SD. (E) The URA3 gene was successfully deleted from the Evo Ura− strain. Whole chromosomes of the Evo strain and 5-fluoroorotic acid (FOA) treated Evo cells (Evo Ura−) were separated by PFGE, stained with ethidium bromide (left) and subjected to Southern hybridization with a URA3 probe to verify successful deletion of all URA3 copies in the genome (right). (F) Phenotype of Evo and Evo Ura− strains after growth for 18 h at 37°C and 5% CO2 in DMEM+10% FBS (representative pictures). Removal of all URA3 copies has no influence on the regained filamentation properties of the Evo strain. (G) Virulence traits of the efg1Δ/cph1Δ (ΔΔ) and Evo strains cured of URA3 (Ura-) and after subsequent re-introduction of a single URA3 copy using the CIp10 plasmid for genomic integration. No difference can be detected between the multi-copy and single-copy strains in adhesion or invasion of epithelial cells, or damage to macrophages. Absence of URA3 reduces macrophage damage, probably due to the low uridine concent [file pgen.1004824.s004.tif]

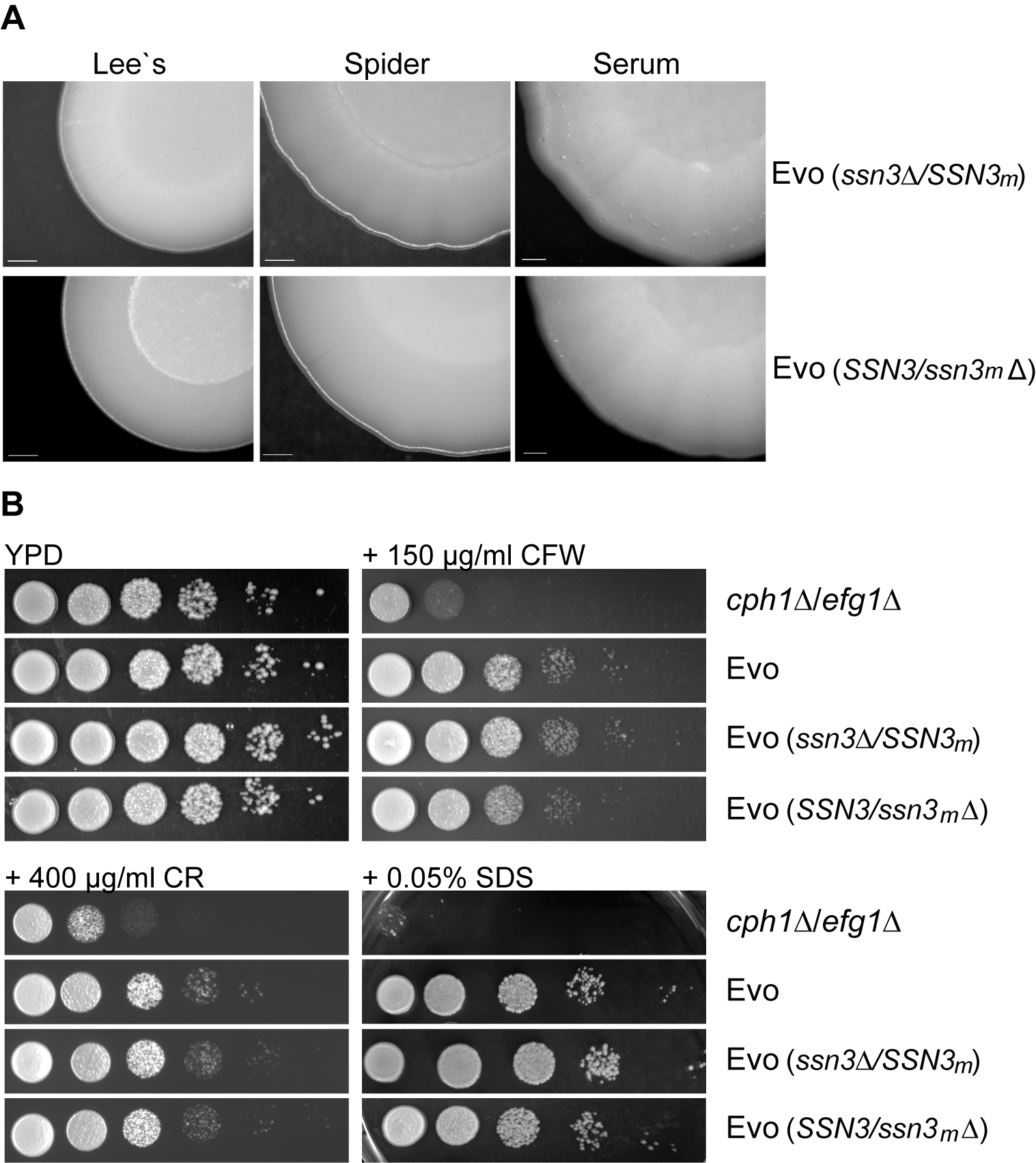

Supplement: S5 Figure — Characterization of the Evo SSN3/ssn3mΔ and Evo ssn3Δ/SSN3m strains. (A) Colony morphology of the Evo ssn3Δ/SSN3m strain grown on solid Lee's medium, Spider medium and YPD medium supplemented with 10% FBS for 6 days at 37°C (scale bar: 1 mm; representative pictures are shown). Observed morphologies resemble those of the Evo strain. (B) Stress resistance of cph1Δ/efg1Δ, Evo, Evo SSN3/ssn3mΔ and Evo ssn3Δ/SSN3m strains against different cell wall perturbing agents. The presence of either SSN3 allele has no influence on the increased stress resistance of the Evo strain. Experiments yielded similar results for at least three replicates. (TIF) [file pgen.1004824.s005.tif]
