# Supplementary material for: Microevolution of Candida albicans in Macrophages Restores Filamentation in a Nonfilamentous Mutant
Source: PLoS Genet. 2014 Dec 4;10(12):e1004824. doi: 10.1371/journal.pgen.1004824 (PMC4256171; doi:10.1371/journal.pgen.1004824)
Supplement: S1 Table — Strains and primers used in the study. (DOC) [file pgen.1004824.s006.doc]

Strains used in this study

| **Strain** | **Genotype** | **Reference** |
| --- | --- | --- |
| **SC5314** | Isogenic wild type | [1] |
| **CAI-4** | *ura3::imm434/ura3::imm434 iro1/iro1::imm434/ RPS10::pCIP10* | [2] |
| **BWP17** | *ura3::imm434/ura3::imm434 iro1/iro1::imm434 his1::hisG/his1::hisG arg4/arg4 RPS10::pCIP30* | [3] |
| ***cph1*Δ/*efg1*Δ** | *CAI-4 cph1Δ::hisG/cph1Δ::hisG/efg1Δ::hisG/efg1Δ::hisG-URA3-hisG* | [4] |
| ***ras1*Δ** | *CAI-4 ras1Δ::hisG/ras1Δ::hisG* | [5] |
| ***rim101*Δ** | *CAI-4 prr2Δ::hisG/prr2Δ::hisG* | [6] |
| ***dfg16*Δ** | *CAI-4 defg16Δ::hisG/dfg16Δ::hisG* | [7] |
| ***tec1*Δ** | *CAI-4 tec1Δ::hisG/tec1Δ::hisG* | [8] |
| ***hgc1*Δ** | *BWP17 hgc1Δ::ARG4/hgc1Δ::HIS1 RPS10::pCIP10* | [9] |
| ***eed1*Δ** | *BWP17 eed1Δ::HIS1/eed1Δ::ARG4 RPS10::pCIP10* | [3] |
| ***ume6*Δ** | *ume6Δ:CdHIS1/ ume6ΔCmLEU2* | [10] |
| ***mkc1*Δ** | *BWP17mkcc1Δ::HIS1/mkc1Δ::ARG4 RPS10::pCIP10* | [11] |
| **Evo** | *CAI-4 cph1Δ::hisG/cph1Δ::hisG/efg1Δ::hisG/efg1Δ::hisG-URA3-hisG* | This study |
| **Evo Ura^-^** | *CAI-4 cph1Δ::hisG/cph1Δ::hisG/efg1Δ::hisG/efg1Δ::hisG* | This study |
| **Evo Ura^-^ *efh1*Δ** | *CAI-4 cph1Δ::hisG/cph1Δ::hisG/efg1Δ::hisG/efg1Δ::hisG/*  *efh1Δ::dpl200/efh1Δ::dpl200-URA3-dpl200* | This study |
| **Evo *ssn3*_m_Δ/*SSN3*** | *CAI-4 cph1Δ::hisG/cph1Δ::hisG/efg1Δ::hisG/efg1Δ::hisG/*  *ssn3_m_::SAT1/SSN3* | This study |
| **Evo *SSN3*_m_/*ssn3*Δ** | *CAI-4 cph1Δ::hisG/cph1Δ::hisG/efg1Δ::hisG/efg1Δ::hisG*  */ssn3::SAT1/SSN3_m_* | This study |
| ***cph1*Δ/*efg1*Δ*_SAT1_*** | *cph1*::FRT/*cph1*::FRT, *efg1*::FRT/*efg1*::FRT | This study |
| ***cph1*Δ/*efg1*Δ*_SAT1_***  ***SSN3*^OE^** | *cph1*::FRT/*cph1*::FRT, *efg1*::FRT/*efg1*::FRT *ADH1*/*adh1*::*SSN3*^WT^-*SAT1* | This study |
| ***cph1*Δ/*efg1*Δ*_SAT1_***  ***SSN3_m_*^OE^** | *cph1*::FRT/*cph1*::FRT, *efg1*::FRT/*efg1*::FRT *ADH1*/*adh1*::*SSN3*^MUT^-*SAT1* | This study |
| ***cph1*Δ/*efg1*Δ*_SAT1_***  ***SSN3_m_*** | *cph1*::FRT/*cph1*::FRT, *efg1*::FRT/*efg1*::FRT *SSN3*/*ssn3*::*SSN3*^MUT^-*SAT1* | This study |

**P**rimers used in this study

| **Name** | **Sequence (5’ – 3’)** | **Reference** |
| --- | --- | --- |
| **qPCR** | | |
| **ACT1-R1** | TCAGACCAGCTGATTTAGGTTTG | [12] |
| **ACT1-R2** | GTGAACAATGGATGGACCAG | [12] |
| **ECE1-R1** | ATCGAAAATGCCAAGAGAG | [12] |
| **ECE1-R2** | AGCATTTTCAATACCGACAG | [12] |
| **EED1-R1** | TAGTGGTAATACCCAACGTG | [12] |
| **EED1-R2** | CTGATATTTGAAATTTTGGAAGCTTTTC | [12] |
| **EFB1-R1** | AGTCATTGAACGAATTCTTGGCTG | [12] |
| **EFB1-R2** | TCTTCATCAACTTCATCATCAGAACC | [12] |
| **HWP1-R1** | ATCAGCTCCTGCCACTGAAC | [12] |
| **HWP1-R2** | TGAGTGGAACTGATTCTAATGTAGTTG | [12] |
| **TEC1-R1** | ACTTGCAACCACACCAAATGTG | [12] |
| **TEC1-R2** | TTCGTGATATTTCCATATCCGGTATTC | [12] |
| **UME6-R1** | TCTACTTCTAATCCAATGGTG | [12] |
| **UME6-R2** | TATCATTACTTGATTTTTTCCGAG | [12] |
| **PMA-fw** | TTGCTTATGATAATGCTCCATACGA | [13] |
| **PMA-re** | TACCCCACAATCTTGGCAAGT | [13] |
| **RT ALS3 for** | TCTCGTCCTCATTACACCAACCAT | [14] |
| **RT ALS3 rev** | GGGGATTGTAAAGTGGATTCTGTG | [14] |
| **BCR1-RT-fw** | CACCTGATCCAAGAGGTTCAA | This study |
| **BCR1-RT-re** | TGAGCCCCAATTAGGATGAG | This study |
| **BRG1-RT-fw** | AGCAGTGCATCAACATCCAG | This study |
| **BRG1-RT-re** | TGCTGCCCCTTTTCTCTTTA | This study |
| **CZF1-RT-fw** | GCAGTGCCAACGTCAGGGTCA | This study |
| **CZF1-RT-re** | CCGCTTCCTGTTGCGCCGT | This study |
| **EFG1-RT-fw** | CCAGTGGTGGCAGTAATGTG | This study |
| **EFG1-RT-re** | TTTTGGTTCTTACCAGGAGCA | This study |
| **EFH1-RT-fw** | GCCACAACAAGATGGGATG | This study |
| **EFH1-RT-re** | AATCCAACCCCCTTCAAAT | This study |
| **NDT80-R-fw** | TGCATGCCCGTATTGATAGA | This study |
| **NDT80-RT-re** | TAGGGTGATGAGGTGGGAAA | This study |
| **ROB1-RT-fw** | TCATTGGTGGCACAAGTATCA | This study |
| **ROB1-RT-re** | TTCATCCCTTCTTCTTTGTTCC | This study |
| **URA3-fw** | GGTGTCACTGGGAATGGAGT | This study |
| **URA3-re** | ACGTTGGGCAATAAATCCAA | This study |
| **SNP validation** | | |
| **19.794-fw** | AACCAATTCCATGTCCTACCA | This study |
| **19.794-re** | TGTTGGTGTCCCCAAGATTT | This study |
| **Strain construction** | | |
| **EFG-A** | GCCTCCCACATTAGTTGCTCAG | [4] |
| **EFG-B** | GAAGATCTGTCCTTGTTGTTGTAGAACAGTAG | [4] |
| **P33** | CGACCTTCCCCCACCTCGTTCCC | [15] |
| **CPH-B** | CCAAAACAGTCAACACAAATAACC | [15] |
| **URA3_2fw** | GGTGTCACTGGGAATGGAGT | [16] |
| **URA3_2re** | ACGTTGGGCAATAAATCCAA | [16] |
| **EFH1_fw** | GTTCCTCACAGTAATAAATAGGAGCTACCCCCAAAATCATTATTTATTAATAATTTGCTTCAAACTATTAATCTCGTTATAAGTATATTCACATATTTTATAAGGTAAAACGACGGCCAGT | This study |
| **EFH1_re** | ATAAATAAACCTAACAATGAATAAAAATAAAATAAATAAAACCAGAATATTTGAAGAAAATTGGCCTTTGAAAAATGTAAACTTTCTTTGTTGTAGTTGTTTAGGAAACAGCTATGACCATG | This study |
| **EFH1_4fw** | CCTGATTTGGAATTCCCTA | This study |
| **EFH1_4re** | TGATTTTGGGGGTAGCTCCT | This study |
| **SSN3_SAT_fw** | TAACTCTTTCTCTCTCTCTCTTTGTCTCTCTTTGTCTCTCACTTTACTTAAAATCAATTCTATTCGTCATTAAGAAAATTACATATAGAAAACCCAATGCAAAT**GAAGCTTCGTACGCTGCAGGTG** | This study |
| **SSN3_SAT_re** | ACGTGTTGTCTGTGAGGATCATGCAGTACCACCACCACCATCAAAATTCAATTCATTTACGATCATTAAAACACTATACAAATAAAAATGACATCTATATCTAT**TCTGATATCATCGATGAATTCGAG** | This study |
| **SSN3_out_fw** | TGCTAATGGTGTCTTTTTGCT | This study |
| **I2-SAT1** | GGCGTCATCCTGTGCTCCCGAG | This study |
| **5'CaSSN3-XhoI** | ATCC**CTCGAG**ATGAGTTATAGTTCAGCTTCATTTAGAAAAC | This study |
| **3’CaSSN3-HincII** | TGTT**GTCGAC**CTACCCACGTTTCTTTCTAATTCCAC | This study |
| **5'CaSSN3term-SacII** | GCCA**CCGCGG**ATAGATATAGATGTCATTTTTATTTGTATAGTGT | This study |
| **3'CaSSN3term-PacISacI** | GCTG**GAGCTCTTAATTAA**TAGCCGTATATATATCAGAAATGGCAC | This study |
| **5'CaSSN3prom-AscI** | TACC**GGCGCGCC**AGTAATATGTTTTTTTAAAAAAAAAAAATGGATATATATATAGAACTATG | This study |
| **ACT1term veri rev** | GAATACAAAACCAGATTTCCAGATTTCCAG | This study |
| **55_CPH1** | ACTTCTGTCCACACACACACTAC | This study |
| **53_CPH1** | ccaattcgccctatagtgagTGGCGAAAGAGTGTATTAGGCAG | This study |
| **55_pSFS3b-CPH1ovup** | CTGCCTAATACACTCTTTCGCCActcactatagggcgaattgg | This study |
| **33_pSFS3b-CPH1ovds** | CTATTCATCTATGTTTGTGACTGtgagcggataacaatttcacac | This study |
| **35_CPH1** | gtgtgaaattgttatccgctcaCAGTCACAAACATAGATGAATAG | This study |
| **33_CPH1** | AAGTAACAACCATCGATATTG | This study |
| **SAT4s** | CTTCGCACTAGTTTCTCGGTACTATG | This study |
| **SAT5s** | CGGGTATTTTCTCTTGTTTGATGATTCATCC | This study |

Underlined nucleotides indicate pDDB57 annealing regions, lower case nucleotides indicate overlaps with NAT1 flipper sequences, bold marked nucleotides indicate pFA annealing regions and bold underlinded nucleotides indicate restriction sites. All primers were obtained from biomers.net GmbH (Ulm, Germany).

**References**

1. Gillum AM, Tsay EY, Kirsch DR (1984) Isolation of the *Candida albicans* gene for orotidine-5'-phosphate decarboxylase by complementation of *S. cerevisiae* *ura3* and *E. coli pyrF* mutations. Mol Gen Genet 198(1): 179-82.

2. Murad AM, Lee PR, Broadbent ID, Barelle CJ, Brown AJ (2000) CIp10, an efficient and convenient integrating vector for *Candida albicans*. Yeast 16(4): 325-7.

3. Zakikhany K, Naglik JR, Schmidt-Westhausen A, Holland G, Schaller M et al. (2007) *In vivo* transcript profiling of *Candida albicans* identifies a gene essential for interepithelial dissemination. Cell Microbiol 9(12): 2938-54.

4. Lo HJ, Kohler JR, DiDomenico B, Loebenberg D, Cacciapuoti A *et al.* (1997) Nonfilamentous *C. albicans* mutants are avirulent. Cell 90(5): 939-49.

5. Feng Q, Summers E, Guo B, Fink G (1999) Ras signaling is required for serum-induced hyphal differentiation in *Candida albicans*. J Bacteriol 181(20): 6339-46.

6. Ramon AM, Porta A, Fonzi WA (1999) Effect of environmental pH on morphological development of *Candida albicans* is mediated via the PacC-related transcription factor encoded by *PRR2*. J Bacteriol 181(24): 7524-30.

7. Thewes S, Kretschmar M, Park H, Schaller M, Filler SG *et al.* (2007) *In vivo* and *ex vivo* comparative transcriptional profiling of invasive and non-invasive *Candida albicans* isolates identifies genes associated with tissue invasion. Mol Microbiol 63(6): 1606-28.

8. Schweizer A, Rupp S, Taylor BN, Rollinghoff M, Schröppel K (2000) The TEA/ATTS transcription factor CaTec1p regulates hyphal development and virulence in *Candida albicans*. Mol Microbiol 38(3): 435-45.

9. Zheng X, Wang Y, Wang Y (2004) Hgc1, a novel hypha-specific G1 cyclin-related protein regulates *Candida albicans* hyphal morphogenesis. Embo J 23(8): 1845-56.

10. Zeidler U, Lettner T, Lassnig C, Müller M, Lajko R *et al.* (2009) *UME6* is a crucial downstream target of other transcriptional regulators of true hyphal development in *Candida albicans*. FEMS Yeast Res 9(1): 126-42.

11. Kumamoto CA (2005) A contact-activated kinase signals *Candida albicans* invasive growth and biofilm development. Proc Natl Acad Sci U S A 102(15): 5576-81.

12. Martin R, Moran GP, Jacobsen ID, Heyken A, Domey J *et al.* (2011) The *Candida albicans*-specific gene *EED1* encodes a key regulator of hyphal extension. PLoS One 6(4): e18394.

13. Nailis H, Coenye T, Van Nieuwerburgh F, Deforce D, Nelis HJ (2006) Development and evaluation of different normalization strategies for gene expression studies in *Candida albicans* biofilms by real-time PCR. BMC Mol Biol 7: 25.

14. Wächtler B, Wilson D, Haedicke K, Dalle F, Hube B (2011) From attachment to damage: defined genes of *Candida albicans* mediate adhesion, invasion and damage during interaction with oral epithelial cells. PLoS One 6(2): e17046.

15. Liu H, Köhler J, Fink GR (1994) Suppression of hyphal formation in *Candida albicans* by mutation of a *STE12* homolog. Science 266(5191): 1723-6.

16. Martin R, Hellwig D, Schaub Y, Bauer J, Walther A *et al.* (2007) Functional analysis of *Candida albicans* genes whose *Saccharomyces cerevisiae* homologues are involved in endocytosis. Yeast 24(6): 511-22.
