# Supplementary material for: Microevolution of Candida albicans in Macrophages Restores Filamentation in a Nonfilamentous Mutant
Source: PLoS Genet. 2014 Dec 4;10(12):e1004824. doi: 10.1371/journal.pgen.1004824 (PMC4256171; doi:10.1371/journal.pgen.1004824)
Supplement: S1 Protocol — Additional methods used in this manuscript and for generation of the supplementary figures. (DOC) [file pgen.1004824.s009.doc]

**Protocol S1**

**Plasmid construction**

For the construction of overexpression plasmids of either wild type or mutant *SSN3*, we used plasmid pSK-ADH1promGFP-SAT1 , containing *CaSAT1* as resistance marker and *ADH1* promoter and terminator sequences for integration into the *ADH1* locus. *GFP* was excised by *Xho*I and *EcoR*V. *SSN3* wild type or mutant alleles were amplified using the primers 5'CaSSN3-XhoI and 3’CaSSN3-HincII. The PCR product was then cloned via XhoI and HincII into the remaining pSK-ADH1prom-SAT1 plasmid. Exchange of GFP by *SSN3* was confirmed by sequencing (GATC Biotech, Konstanz, Germany). For transformation into *C. albicans*, ADH1prom-SSN3-SAT1 was excised by *Asc*I and *Pac*I.

To integrate the mutated *SSN3* allele into the native *SSN3* locus, the *ADH1* terminator was excised from pSK-ADH1promGFP-SAT1 by *Sac*II and *Sac*I. Primers 5'CaSSN3term-SacII and 3'CaSSN3term-PacISacI were used to amplify the *SSN3* promoter from genomic *C. albicans* DNA. This was cloned into the linearized pSK-ADH1promGFP-SAT1 plasmid. From this pSK-ADH1prom-GFP-SAT1-SSN3term plasmid, the *ADH1* promoter and *GFP* were excised by *Asc*I and *Hinc*II and a PCR product of *SSN3* promoter and *SSN3_m_* ORF (amplified with 5'CaSSN3prom-AscI and 3'CaSSN3-HincII) was integrated in its place. The resulting plasmid pSK-SSN3_m_-SAT1 was confirmed by sequencing (GATC Biotech, Konstanz, Germany). For transformation, *SSN3-SAT1* was excised by *Asc*I and *Pac*I.

**Strain construction**

The uridine auxotroph Evo mutant (Evo Ura^-^) was obtained by plating the strain on 0.1% 5‑fluoroorotic acid (5-FOA) containing SD plates. The removal of *URA3* from the *EFG1* locus was verified by southern blot analyses as described previously and in short in the section “Evolution experiment”. Given that the evolved strain exhibit a multiplication of *URA3*, southern blot analyses using a *URA3* specific probe, generated with the primers URA3_2fw and URA3_2re, was performed on gDNA digested with *Psi*I or *Hind*III and *Nco*I, respectively, as well as on PFGE resolved chromosomes.

C. albicans deletion mutants were generated using a PCR product-directed disruption technique in combination with the lithium-acetate transformation method as described previously . To generate the Evo Ura^-^ (*efh1*Δ/*efh1*Δ) mutant the complete open reading frame of one *EFH1* allele was replaced with a PCR-amplified *URA*3-*dpl200* deletion cassette flanked by 104 bp of target homolgy region, in a first round of deletion. Next, positive transformants were selected for loss of *URA3* by plating them on 5-FOA medium. The *URA3*-*dpl200* disruption cassette was used again to disrupt the second allele. Primers EFH1_fw and EFH1_re were used for the generation of the *URA3*-*dpl200* deletion cassette with the pDDB57 plasmid . The correct deletion of both alleles was verified by southern blot analysis using a PCR product, generated with the primers EFH1_4fw and EFH1_4re from *C. albicans* SC5314 gDNA as a probe on *Ale*I and *EcoR*I-digested gDNA. To generate the Evo *SSN3*/*ssn3_m_*Δ and Evo *ssn3*Δ*/SSN3_m_* mutants, respectively, PCR products used in transformation were amplified from the pFA cassette using the primers SSN3_SAT_fw and SSN3_SAT_re. Within these primers, 104 nucleotides of the target homology region are incorporated at their 5′ ends. Transformants were grown on YPD plates containing 200 µg/ml nourseothricin (NAT) and incubated for one‑two days at 30°C. Verification of the correct integration at the *SSN3* locus was done by PCR using the flanking primer SSN3_out_fw and the cassette internal primer I2-SAT1. Next, presence of either the mutated or the wild type *SSN3* allele was validated by Sanger sequencing using primers 19.794‑fw and 19.794‑re. PCR was performed with Phusion HighFidelity DNA polymerase (NEB). Purified products (QIAquick Gel Extraction Kit, Qiagen) were sequenced by GATC Biotech (Konstanz, Germany). BioEdit version 7.0.5.3 was used to analyze the sequences. All primers are listed in Table S1.

The *cph1*Δ/*efg1*Δ*_SAT1_* strain is based on the clinical isolate SC5314. All homozygous gene deletions for this strain were done with a modified SAT1-flipper technique . The *EFG1* mutant was described earlier . For *cph1*Δ/*efg1*Δ*_SAT1_*, a *CPH1* deletion cassette was constructed by fusion PCR: 400 to 500 bp flanks upstream and downstream of the *CPH1* were PCR amplified from SC5314 gDNA using primers 55_CPH1 & 53_CPH1 and 35_CPH1 & 33_CPH1. The NAT1-flipper was PCR amplified from plasmid pSFS3b using the primers 55_pSFS3b-CPH1ovup and 33_pSFS3b-CPH1ovds. First, the *CPH1* upstream and downstream regions were fused to the NAT1-flipper using the primers 55_CPH1, SAT5s and 33_CPH1, SAT4s, respectively. Those two fragments were then fused together using the primers 55_CPH1 and 33_CPH1 to yield the *CPH1* deletion cassette. This cassette was transformed into the *EFG1* deletion strain via electroporation, and correct genomic integration was confirmed by colony PCR and Southern Blot.

To generate *cph1*Δ/*efg1*Δ*_SAT1_SSN3*^OE^, *cph1*Δ/*efg1*Δ*_SAT1_SSN3*_m_^OE^ and *cph1*Δ/*efg1*Δ*_SAT1_SSN3*_m_, *cph1*Δ/*efg1*Δ*_SAT1_* was transformed with the corresponding plasmids using the lithium acetate protocol. After the heat shock, cells were grown in YPD for 4h at 30°C and then plated on YPD with 200 µg/ml NAT. Plates were then further incubated for 2 to 3 days at 30°C. Verification of the transformants was performed by colony PCR using the primers 19.794-fw and ACT1term veri rev.

**Phenotypic characterization**

To investigate filament formation in liquid media, 1×10^4^ yeast cells/well were incubated in a 24 well microtiter plate comprising DMEM + 10% FBS, YPD + 10% FBS, H_2_O + 10% FBS or GlcNac medium (1 x YNB without amonium sulfate, 2% sucrose, 75 mM MPOS, 5 mM GlcNac [Sigma]) at 37°C and 5% CO_2_, unless indicated otherwise. Filamentous growth induction on solid media was carried out by spotting 1×10^5^ cells either on Spider medium , Lee’s medium , serum‑containing YPD medium, YNB (1.7% yeast nitrogen base without amino acids and ammonium sulfate, supplemented with 2% glucose) medium containing the alternative nitrogen source urea or by embedding in YPS agar (1% yeast extract, 2% bacto-peptone, 2% sucrose, 1% agar) and incubated at 37°C except for embedded plates, which were incubated at 23°C. The morphology index of cells incubated in DMEM + 10% FBS at 37°C and 5% CO_2_ was calculated as previously described . In experiments using farnesol (Sigma),a 100 mM stock solution was prepared in methanol. For cAMP experiments, a 100 mM stock solution was prepared in water and added immediately after the farnesol. Growth in the presence of 450 µg/ml CR, 200 µg/ml calcofluor white (CFW) and 0.05% SDS was examined by spotting 5 µl of different dilutions of overnight cultures covering a range of 10^6^‑10^1^ cells onto YPD plates with added, different stressors. Plates were incubated at 37°C for two days and documented by photography.

**Staining procedures and detection of β-1,3-glucans and mannans**

For filipin (Sigma) staining, 1 mg/ml filipin was dissolved in DMSO and the working concentration of 10 µg/ml was prepared in PBS. Cells were stained for 20 min at room temperature and directly analyzed by fluorescence microscopy. All subsequent washing steps were done with sterile PBS. Prior CFW and Als3 staining, fungal cells were fixed with 4% paraformaldehyde. *C. albicans* cells were subsequently stained with 100 µg/ml CFW (diluted in 100 mM Tris/HCl pH 9.0) for 15 min at room temperature. After washing with destilled water, cells were analyzed by fluorescence microscopy. To visualize the surface expression of Als3, the cells were treated with a rabbit polyclonal antiserum raised against the recombinant N-terminal region of Als3 as described before . Flow cytometry was used to quantify mannan and β‑1,3-glucan exposure on the surface of stationary *C. albicans* cells by using concanavalin A – Alexa 647 (ConA; Molecular Probes) and the monoclonal antibody to β‑1,3-glucan (mouse IgG; Biosupplies). For ConA staining cells were washed twice and fixed with 4% paraformaldehyde. Next, cells were washed twice and 1×10^6^ cells were stained with 10 µg/ml ConA (in sterile PBS) for 30 min with gentle shaking in the dark. After three washing steps the fluorescence intensity was measured. For β‑1,3-glucan staining 1×10^8^ paraformaldehyde-fixed cells were washed twice, followed by blocking with 1% BSA for one hour at room temperature with gentle shaking. A 1:300 dilution of anti- β‑1,3-glucan in 1% BSA was used as the primary antibody and cells were incubated over night at 4°C with gentle shaking. After three washing steps cells were incubated in a 1:600 dilution of goat anti-mouse IgG Alexa Fluor 488 (Molecular Probes) as second antibody for one hour at room temperature with gentle shaking in the dark. Subsequently, the cells were washed 3 times and fluorescence intensity was quantified. Differential staining of extra- and intracellular fungal cell parts was performed as previously described . In brief, extracellular parts were stained in fixed samples by Alexa Fluor 488 conjugate concanavalin A (Invitrogen). After permeabilization with 0.5% Triton X‑100, the complete fungal cells were stained with CFW.

**Pulsed-ﬁeld gel electrophoresis (PFGE) and SNP-RFLP analysis**

To prepare samples for PFGE cells of an overnight culture were washed two times with water and protoplast were generated by incubating the cells in 0.8 M NaCl supplemented with 500 µg/ml zymolyase 20T (amsbio) for one hour at 37°C with gentle shaking. Protoplasts were centrifuged for 10 min at 1000 g and kept in 0.8 M NaCl. At 40°C pre‑warmed protoplasts suspension was mixed with Low melt Agarose (PeqLab) at a 3:5 ratio. Plugs were poured immediately and placed at 4°C for 20 min. Next, agarose plugs were carefully removed and incubated for one hour in lysisbuffer (0.5 M EDTA pH 9.5, 0.1 M Tris, 1% N-lauroyl sarcosinate) followed by an incubation in lysisbuffer supplemented with 2 mg/ml proteinase K (AppliChem) for 16‑18 h at 37°C. Plugs were stored in 0.5 M EDTA pH 9.5 at 4°C until use. Prior PFGE agarose plugs were incubated in 0.5xTBE buffer pH 8.2 for 12 h. Plugs were placed in the wells of a 1% Biozym Gold Agarose (Biozym) gel and wells were sealed with low melt agarose. The gel was run in a CHEF-DR II system (BioRad) in 0.5xTBE buffer and chromosomes were separated using the following conditions: 24 h with a 120 s switch followed by 40 h with a 240 s switch at 8 V/cm at 10°C. . Gels were stained with ethidium bromide for 30 min and destained in running buffer for 2 h, prior to image acquisition. SNP‑RFLP analysis was performed to detect short‑tract, long‑tract and whole chromosome LOH events. 32 SNP‑RFLP markers, 4 per chromosome (2 markers per chromosome arm) were analyzed as described previously . Briefly, PCRs using 30 ng gDNA and SNP-RFLP-specific primer pairs (listed in Table S7 in ) were performed in 96‑well microtiter plate format, and five µl of each PCR product were digested over night with the appropriate restriction enzyme. Digested PCR products were separated in a 3% agarose gel (with ethidium bromide) along with undigested and digested controls, gels were photographed, and restriction patterns were analyzed.

**References**

1. Hünniger K, Lehnert T, Bieber K, Martin R, Figge MT, et al. (2014) A virtual infection model quantifies innate effector mechanisms and *Candida albicans* immune escape in human blood. PLoS Comput Biol 10: e1003479.

2. Lo HJ, Köhler JR, DiDomenico B, Loebenberg D, Cacciapuoti A, et al. (1997) Nonfilamentous *C. albicans* mutants are avirulent. Cell 90: 939-949.

3. Gola S, Martin R, Walther A, Dünkler A, Wendland J (2003) New modules for PCR-based gene targeting in *Candida albicans*: rapid and efficient gene targeting using 100 bp of flanking homology region. Yeast 20: 1339-1347.

4. Walther A, Wendland J (2003) An improved transformation protocol for the human fungal pathogen *Candida albicans*. Curr Genet 42: 339-343.

5. Wilson RB, Davis D, Enloe BM, Mitchell AP (2000) A recyclable *Candida albicans* *URA3* cassette for PCR product-directed gene disruptions. Yeast 16: 65-70.

6. Schaub Y, Dünkler A, Walther A, Wendland J (2006) New pFA-cassettes for PCR-based gene manipulation in *Candida albicans*. J Basic Microbiol 46: 416-429.

7. Tscherner M, Stappler E, Hnisz D, Kuchler K (2012) The histone acetyltransferase Hat1 facilitates DNA damage repair and morphogenesis in *Candida albicans*. Mol Microbiol 86: 1197-1214.

8. Hnisz D, Schwarzmüller T, Kuchler K (2009) Transcriptional loops meet chromatin: a dual-layer network controls white-opaque switching in *Candida albicans*. Mol Microbiol 74: 1-15.

9. Liu H, Köhler J, Fink GR (1994) Suppression of hyphal formation in *Candida albicans* by mutation of a *STE12* homolog. Science 266: 1723-1726.

10. Lee KL, Buckley HR, Campbell CC (1975) An amino acid liquid synthetic medium for the development of mycelial and yeast forms of *Candida albicans*. Sabouraudia 13: 148-153.

11. Merson-Davies LA, Odds FC (1989) A morphology index for characterization of cell shape in *Candida albicans*. J Gen Microbiol 135: 3143-3152.

12. Phan QT, Myers CL, Fu Y, Sheppard DC, Yeaman MR, et al. (2007) Als3 is a *Candida albicans* invasin that binds to cadherins and induces endocytosis by host cells. PLoS Biol 5: e64.

13. Dalle F, Wächtler B, L'Ollivier C, Holland G, Bannert N, et al. (2010) Cellular interactions of *Candida albicans* with human oral epithelial cells and enterocytes. Cell Microbiol 12: 248-271.

14. Forche A, Steinbach M, Berman J (2009) Efficient and rapid identification of *Candida albicans* allelic status using SNP-RFLP. FEMS Yeast Res 9: 1061-1069.
